# Functional analysis of the *Helicobacter pullorum N*-linked protein glycosylation system

**DOI:** 10.1093/glycob/cwx110

**Published:** 2018-01-11

**Authors:** Adrian J Jervis, Alison G Wood, Joel A Cain, Jonathan A Butler, Helen Frost, Elizabeth Lord, Rebecca Langdon, Stuart J Cordwell, Brendan W Wren, Dennis Linton

**Affiliations:** 2 Manchester Institute of Biotechnology, SYNBIOCHEM, University of Manchester, Manchester, UK; 3 Department of Molecular Biology and Biotechnology, University of Sheffield, Sheffield, UK; 4 School of Molecular Bioscience and Charles Perkins Centre, The University of Sydney, Australia; 5 School of Healthcare Science, Manchester Metropolitan University, Manchester, UK; 6 Faculty of Biology, Medicine and Health, Michael Smith Building, University of Manchester, Manchester, UK; 7 Pathogen Molecular Biology Unit, London School of Hygiene and Tropical Medicine, London, UK

**Keywords:** bacteria, glycoprotein, glycosylation, Helicobacter, *N*-linked

## Abstract

*N*-linked protein glycosylation systems operate in species from all three domains of life. The model bacterial *N*-linked glycosylation system from *Campylobacter jejuni* is encoded by *pgl* genes present at a single chromosomal locus. This gene cluster includes the *pglB* oligosaccharyltransferase responsible for transfer of glycan from lipid carrier to protein. Although all genomes from species of the *Campylobacter* genus contain a *pgl* locus, among the related *Helicobacter* genus only three evolutionarily related species (*H. pullorum*, *H. canadensis* and *H. winghamensis*) potentially encode *N*-linked protein glycosylation systems. *Helicobacter* putative *pgl* genes are scattered in five chromosomal loci and include two putative oligosaccharyltransferase-encoding *pglB* genes per genome. We have previously demonstrated the in vitro *N*-linked glycosylation activity of *H. pullorum* resulting in transfer of a pentasaccharide to a peptide at asparagine within the sequon (D/E)XNXS/T. In this study, we identified the first *H. pullorum N*-linked glycoprotein, termed HgpA. Production of histidine-tagged HgpA in the background of insertional knockout mutants of *H. pullorum pgl*/*wbp* genes followed by analysis of HgpA glycan structures demonstrated the role of individual gene products in the PglB1-dependent *N*-linked protein glycosylation pathway. Glycopeptide purification by zwitterionic-hydrophilic interaction liquid chromatography coupled with tandem mass spectrometry identified six glycosites from five *H. pullorum* proteins, which was consistent with proteins reactive with a polyclonal antiserum generated against glycosylated HgpA. This study demonstrates functioning of a *H. pullorum N*-linked general protein glycosylation system.

## Introduction

In all three domains of life subsets of proteins are modified by the covalent attachment of sugars to asparagine residues within a conserved consensus sequon of N-X-S/T (Calo et al. 2010; Nothaft and Szymanski 2010; Larkin and Imperiali 2011; Schwarz and Aebi; 2011; Eichler 2013). Among Bacterial species, two such distinct *N*-linked glycosylation systems have been identified. The first involves attachment of monosaccharide to asparagine by a cytoplasmic *N*-glycosyltransferase and has been characterized in *Haemophilus influenzae* (Grass et al. 2003, 2008, 2010) and *Actinobacillus pleuropneumoniae* (Choi et al. 2010; Kawai et al. 2011; Schwarz et al. 2011a; Naegeli et al. 2014; Cuccui et al. 2017), with further *N*-glycosyltransferase orthologues identified in a number of other species including pathogenic *Yersinia* spp., and enterotoxigenic *Escherichia coli* (Grass et al. 2010). The second type of bacterial *N*-linked protein glycosylation system was discovered in *Campylobacter jejuni* (Szymanski et al. 1999) and subsequently other related species from the Epsilon subdivision of the Proteobacteria (Nothaft and Szymanski 2010). In this system cytoplasmic assembly of an oligosaccharide on an isoprenoid lipid, is followed by transfer across the inner membrane and attachment onto proteins in the periplasm mediated by an integral membrane oligosaccharyltransferase (OTase). The prototypical OTase-dependent *N*-linked protein glycosylation system of *C. jejuni* has been intensively studied. More than 60 extracytoplasmic proteins are known to be glycosylated within an extended N-X-S/T sequon containing an acidic residue (D/E) at the −2 position (Kowarik et al. 2006; Wacker et al. 2006; Chen et al. 2007; Gerber et al. 2013) although examples of nonclassical occupied sequons (without the D/E at the −2 position or S/T at the +2 position) have also been demonstrated (Scott et al. 2014). A single locus contains genes required for biosynthesis, transport and linkage of a conserved heptasaccharide to protein. Five cytoplasmic glycosyltransferases (PglA, PglC, PglJ, PglH and PglI) assemble the heptasaccharide on the lipid carrier (Glover et al. 2005a; Linton et al. 2005; Glover et al. 2006). This is transported across the inner-membrane and into the periplasm by the “flippase” PglK (Alaimo et al. 2006) and transferred onto protein by the OTase, PglB (Wacker et al. 2002; Glover et al. 2005b; Lizak et al. 2011). Three further proteins (PglD, PglE and PglF) are required for biosynthesis of the reducing end sugar, a diacetamidotrideoxyhexose known as di-*N*-acetyl bacillosamine (diNAcBac), from *N*-acetyl glucosamine (Olivier et al. 2006). The *C. jejuni pgl* gene locus when expressed in *E. coli* results in *N*-linked protein glycosylation (Wacker et al. 2002) and can be used to glycosylate an array of target proteins with diverse glycan (Feldman et al. 2005; Iwashkiw et al. 2012; Jervis et al. 2012). The specificity of the *C. jejuni* PglB for lipid-linked oligosaccharides (LLOs) with an acetamido group on the C-2 carbon of the reducing end sugar and accessibility of the target sequon on the surface of the folded target protein, are the major limitations of this approach to generating *N*-linked glycoproteins of choice.

**Fig. 1.**
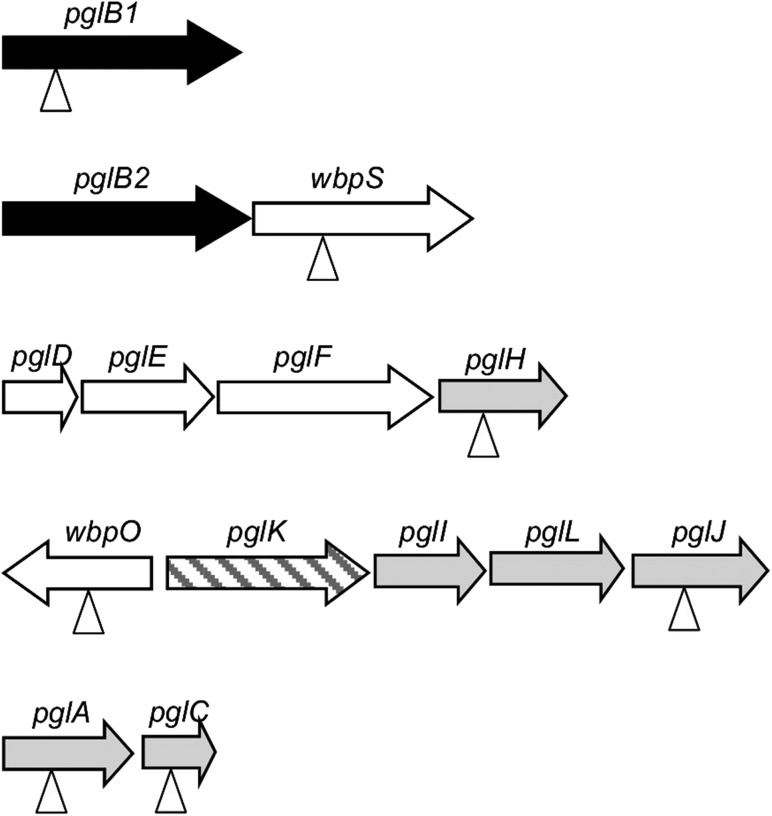
Schematic representation of the fragmented *H. pullorum pgl* gene loci. Individual coding sequences are represented by horizontal arrows and gene designations are based on significant levels of sequence similarity to *C. jejuni pgl* genes (*pglABCDEFHIJK*) or *Pseudomonas aeruginosa wbp* genes (*wbpOS*). The putative gene labeled *pglL* encodes an as yet uncharacterized glycosyltransferase. The genes encoding putative *N*-linked OTases or PglB proteins are shaded black, those encoding putative sugar biosynthesis enzymes are unshaded, those encoding glycosyltransferases are lightly shaded, and the putative transporter or flippase encoding *pglK* is striped. Vertical arrowheads indicate genes that were disrupted through insertion of a kanamycin resistance cassette with the same transcriptional polarity as the mutated gene and characterized herein.

Characterization of further bacterial PglBs has led to identification of OTases from many *Campylobacter* species (Jervis et al. 2012; Nothaft et al. 2012), *Desulfovibrio desulfuricans* (Ielmini and Feldman 2011) and deep sea vent dwelling organisms *Nitratiruptor tergarcus*, *Sulfurovum lithotrophicum* and *Deferribacter desulfuricans* with some displaying differing sequon recognition and glycan promiscuity (Mills et al. 2016). In addition to *pglB* genes, *pgl* gene-containing genetic loci are present in the *Campylobacter* species genomes sequenced to data (Nothaft and Szymanski 2010), and the structures of many of the corresponding *N*-linked glycans were characterized (Jervis et al. 2012; Nothaft et al. 2012). Although the genera *Campylobacter* and *Helicobacter* are closely related, orthologues of the *Campylobacter pgl* genes are absent in genomes of most *Helicobacter* species, including *Helicobacter pylori*. However, *pgl* gene orthologues are present in a single evolutionarily related group of three *Helicobacter* species: *Helicobacter pullorum*, *Helicobacter canadensis* and *Helicobacter winghamensis* (Jervis et al. 2010). In contrast to *Campylobacter* species, the Helicobacter *pgl* genes are scattered around five loci (Figure 1). A further significant deviation from the *C. jejuni* model is the presence in these *Helicobacter* species of not one but two *pglB* genes potentially encoding distinct putative *N*-linked OTases. In our previous work we have demonstrated that a *H. pullorum* membrane extract is capable of in vitro *N*-linked peptide glycosylation with a linear pentasaccharide glycan consisting of HexNAc-216-217-217-HexNAc where 216 and 217 represent the mass differences between species generated by glycan fragmentation and correspond to residues with MH+ values of 217 and 218 Daltons, respectively. Peptide *N*-glycosylation was *H. pullorum pglB1* dependent and required an acidic residue at the −2 position of the sequon as for *C. jejuni* (Jervis et al. 2010). In this more in-depth study, we have demonstrated the in vivo functioning of a *H. pullorum* PglB1-dependent *N*-linked general protein glycosylation pathway.

## Results

### *H. pullorum N*-linked protein glycosylation loci

**Fig. 2.**
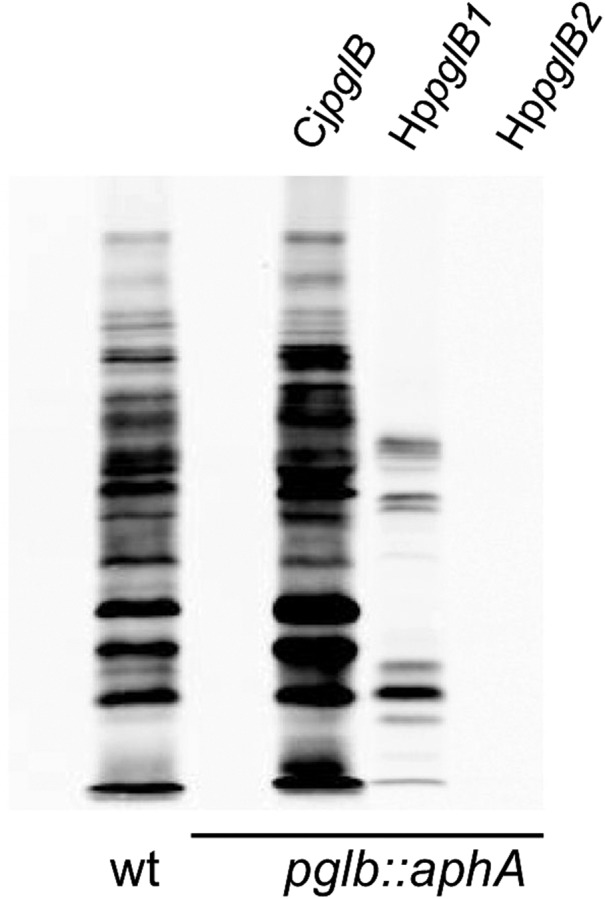
Assay for *N*-linked protein glycosylation activity in *C. jejuni*. Western blotting of *C. jejuni* whole-cell extracts with hR6 glycan-specific antiserum was used to investigate activity of the *H. pullorum* OTases HpPglB1 and HpPglB2. As previously observed wild-type (wt) *C. jejuni* produces a large number of hR6-reactive bands and this immunoreactivity is completely abolished by insertional mutagenesis of *pglB* (*pglB::aphA*). Immunoreactivity was restored through complementation with an introduced chromosomal copy of *C. jejuni pglB* (Cj*pglB*). More limited hR6 reactivity was restored with *H. pullorum pglB1* (Hp*pglB1*) but not *pglB2* (Hp*pglB2*).

Orthologues of *C. jejuni pglABCDEFHIJK* genes that encode the well-characterized *N*-linked protein glycosylation system are present in *H. pullorum* (Figure 1) as well as the closely related species *H. canadensis* and *H. winghamensis* but not in other *Helicobacter* species. These *Helicobacter* species also possess a putative glycosyltransferase-encoding gene located between *pglI* and *pglJ* that is absent in *C. jejuni* and is here designated *pglL* (Figure 1). Two further genes co-located with Helicobacter *pgl* genes also lack *C. jejuni* orthologues. Their predicted products have significant levels of sequence similarity to WbpOS enzymes involved in sugar biosynthesis (King et al. 2010) and we have thus named them *wbpO* and *wbpS* (Figure 1). In contrast to *C. jejuni* and the majority of *Campylobacter* species where *pgl* genes are located in a single locus, in *Helicobacter* species these genes are present in five distinct loci. A notable feature of the Helicobacter *pgl* gene loci is the presence of two orthologues (*pglB1* and *pglB2*) of the single Campylobacter *pglB* gene encoding the OTase (Figure 1). Amino acid sequence alignment of the *Helicobacter* PglBs with the structurally and mechanistically characterized PglB of *Campylobacter lari* and the well-characterized *C. jejuni* PglB showed a high degree of conservation of the known essential residues for oligosaccharyltransferase activity. Catalytically active residues D_54_, R_145_, D_152_, D_154_, E_316_ and R_372_ and the _455_WWD_457_ motif required for peptide binding (Lizak et al. 2011, 2013; Gerber et al. 2013) are all absolutely conserved in the *Helicobacter* PglB1 and PglB2 enzymes (Figure S1). This strongly indicates both PglB1 and PglB2 possess oligosaccharyltransferase or related activity with potentially two distinct *N*-linked protein glycosylation systems operating in *H. pullorum*. To investigate *N*-linked protein glycosylation in this species we first determined whether both *pglB1* and *pglB2* genes were expressed. Specific intragenic primers were designed for both genes (Table SI) and RT-PCR used to detect corresponding transcripts. The generation of RT-PCR products of the predicted sizes (Figure S2) indicates both genes were transcribed during in vitro growth. To demonstrate *N*-linked OTase activity of *H. pullorum* PglBs we expressed both genes in the background of a *C. jejuni pglB* insertional knockout mutant (*pglB*::*aphA*). In this mutant, *N*-linked glycoproteins are not produced and we propose that the presence of lipid-linked heptasaccharide and numerous sequon-containing target proteins provides a sensitive and convenient assay for detecting related *N*-linked OTase activities. The *pglB* genes were recombined onto the *C. jejuni pglB*::*aphA* chromosome within pseudogene Cj0223 (see Methods), and complementation in this way with the *C. jejuni pglB* gene fully restored glycosylation as detected by reactivity of numerous proteins with the *N*-linked heptasaccharide specific antiserum hR6 (Figure 2). Complementation with the *H. pullorum pglB1* gene also restored hR6 immunoreactivity though relatively few proteins were glycosylated (Figure 2). In contrast, the *pglB2* gene did not restore detectable levels of hR6 immunoreactivity (Figure 2). These data confirm that *pglB1* encodes an *N*-linked OTase able to transfer the *C. jejuni* heptasaccharide glycan onto protein whilst the activity of PglB2 remains elusive.

### Identification of *H. pullorum N*-linked glycoproteins

**Fig. 3.**
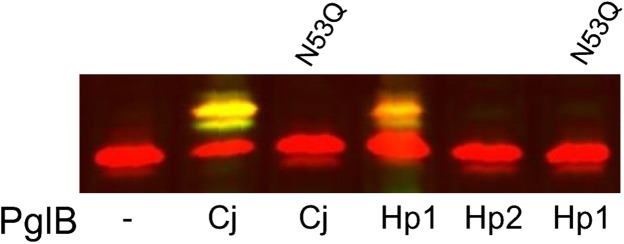
HgpA glycosylation in *E. coli* by *C. jejuni* and *H. pullorum* PglBs. The HgpAhis protein was detected using an anti-his antibody (red), and *C. jejuni N*-linked heptasaccharide glycan detected with hR6 antiserum (green). When *H. pullorum* HgpAhis was produced in *E. coli* a lower mobility form was observed in cells co-expressing either *C. jejuni pglB* (Cj) or *H. pullorum pglB1* (Hp1). This lower mobility form was labeled yellow due to reactivity with both anti-his (red) and anti-glycan (green) antisera indicating *N*-linked glycosylation of HgpAhis. The lower mobility form was absent in cells expressing *H. pullorum pglB2* (Hp2). Production of glycosylated HgpAhis by *C. jejuni* PglB and *H. pullorum* PglB1 was abolished by conversion of the asparagine at residue 53 to a glutamine as indicated.

To directly demonstrate activity of the *H. pullorum N*-linked protein glycosylation system we sought to identify corresponding *N*-linked glycoproteins. Initial efforts to identify a lectin that interacts with such glycoproteins were unsuccessful (data not shown). We therefore undertook an unbiased approach based on glycopeptide enrichment and site-specific identification using MS/MS. Whole cell protein lysates were digested with trypsin and glycopeptides enriched using ZIC-HILIC prior to identification by CID MS/MS to provide *N*-glycan structural information and HCD MS/MS to identify the peptide backbone (Scott et al. 2011). We identified 62 glycopeptides modified with the HexNAc-216-217-217-HexNAc pentasaccharide (Figure S3, Table SIV), which represent six confirmed sites of *N*-glycosylation from five *H. pullorum* proteins (Table I). These proteins are predominantly of unknown function, however all are predicted periplasmic or membrane-associated proteins. Given that many *C. jejuni N*-linked glycoproteins have now been identified (Scott et al. 2011), we searched the five confirmed *H. pullorum* glycoprotein sequences against the *C. jejuni* NCTC 11168 genome. *C. jejuni* contained an orthologue for all five *H. pullorum* glycoproteins (Table I), with sequence identities of between 24.0% and 38.0% (data not shown). Examination of the literature confirmed that four of the five orthologues are known *C. jejuni* glycoproteins, with only Cj1259 as an unknown *N*-glycoprotein. Cj1259 is the major outer membrane protein (PorA or MOMP) in *C. jejuni* and the NCTC 11168 sequence contains no *N*-linked sequons. MOMP has however, recently been identified as a unique *O*-glycoprotein modified with a four residue glycan at a single threonine residue (Mahdavi et al. 2014). We additionally noted that *H. pullorum* glycoprotein Hp00510 is an orthologue of the *C. jejuni N*-linked glycoprotein A or CgpA (Wacker et al. 2002). We therefore named this protein HgpA (Helicobacter glycoprotein A). HgpA is a predicted periplasmic protein with a single *N*-linked glycosylation sequon of ENNDT and is annotated as HPMG_01281 in the *H. pullorum* MIT 98-5489 genome sequence. To further investigate *N*-linked glycosylation of HgpA, the corresponding gene was cloned and expressed in *E. coli* from plasmid pQEhgpA (Table SII and Methods) along with the *C. jejuni pgl* locus on a second plasmid (Wacker et al. 2002). Western blotting with *C. jejuni N*-linked heptasaccharide glycan-specific antiserum hR6 demonstrated that HgpA was glycosylated in a *C. jejuni* PglB-dependent manner (Figure 3). The HgpA protein was similarly glycosylated in *E. coli* by *H. pullorum* PglB1, but not PglB2 (Figure 3). Glycosylation of HgpA by both *C. jejuni* PglB and *H. pullorum* PglB1 was dependent on presence of the asparagine (underlined) within the ENNDT *N*-linked glycosylation sequon identified above (Figure 3).

**Table I.** Identification of glycopeptides from *H. pullorum* using ZIC-HILIC and LC–MS/MS

Gene number Hp. No. (Cj. No.)	Protein identification	Protein mass (Da)	Precursor mass (Charge)	MASCOT score^a^	Peptide sequence^b^
Hp00296c (Cj0114)	Tetratricopeptide Repeat Protein	35,661	1888.8647 (3+)	69.87	^160^KDTIKEDSV*EN**N**GS*APNA^177^
			2371.1319 (3+)	27.25	^175^PNANANIATIESAEN**N**QKESKQ^196^^c^
Hp00314c (Cj1565c)	Flagellar Functional Protein	93,678	4634.1816 (6+)	46.17	^427^AHHYYQMLLQNPKDEAEEKEIQALDDTLLLNYE*DD**N**AT*K^465^
Hp00510 (Cj1670c)	Hypothetical Protein (HpgA)	28,021	1896.9534 (3+)	56.58	^44^KEIPTPN*EN**N**DT*KEIR^59^
Hp00561c (Cj0633)	Hypothetical Protein	38,588	4941.5575 (6+)	37.78	^68^TLQQ*ENNQT*SQSTIPQITPPTISQESKPTKPTQIPSKPKPQCQK^111^
Hp01062c (Cj1259)	Putative Membrane Protein	56,457	1447.6162 (3+)	63.45	^87^DRVDNGNG*DV**N**GS*K^100^

^a^MASCOT score represents the best score provided for the identified glycosylation sequon.

^b^Sequon is shown in italics, with glycosylation site in bold and underlined.

^c^Full sequon was not identified in this peptide and the underlined Asn is predicted only.

### Further characterization of HgpA N-linked glycosylation

A *C*-terminal deca-histidine-tagged version of *hgpA* (*hgpA*his) was recombined onto the *H. pullorum* NCTC 12824 chromosome via plasmid pHPC2hgpAhis (see Methods) to produce strain Hp47 (Table SIII). In order to verify that HgpAhis_10_ was glycosylated, it was purified by nickel affinity chromatography (see Methods). ZIC-HILIC enrichment and MS/MS of tryptic peptides from HgpAhis_10_ identified a glycopeptide with mass (MH+) of 4208.96 Da. CID MS/MS confirmed the presence of the pentasaccharide glycan (Figure S4a) while HCD MS/MS identified the peptide containing the previously identified sequon ENNDT (Figure S4b). HCD MS/MS also shows intense singly charged oxonium ions at 204.08 (HexNAc), 217.08 and 218.07 *m*/*z*. Elemental composition analysis suggests peaks with monoisotopic masses of 217.08190 and 218.06591 likely correspond to C_8_H_13_N_2_O_5_ and C_8_H_12_N_1_O_6_, respectively (Figure S4c). These compositions are consistent with those determined for the uronamide HexNAcAN and the uronate HexNAcA. Overall these data suggest a *H. pullorum N*-linked pentasaccharide with the structure HexNAc–HexNAcAN–HexNAcA–HexNAcA–HexNAc.

### Identification of *H. pullorum* genes involved in HgpA *N*-linked glycosylation

**Fig. 4.**
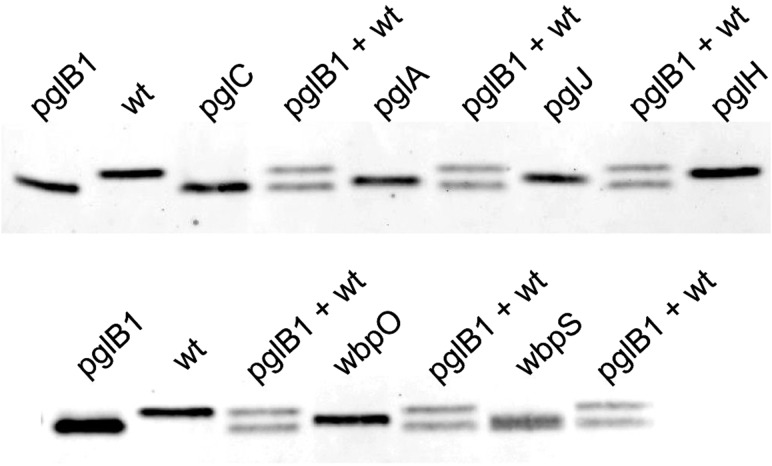
Insertional knockout mutagenesis of genes from five distinct *H. pullorum* genetic loci results in increased mobility of glycoprotein HgpA. A C-terminal histidine tagged version of the HgpA protein was produced in *H. pullorum* wild type and mutant backgrounds (as indicated above lanes) through integration of the corresponding gene onto the chromosome (see Methods). Following SDS-PAGE of whole-cell lysates and western blotting, HgpAhis was detected with anti-his antibody. A single band of increased (relative to that produced in the wild type strain) but varying mobility was observed in individual mutants. For comparison, extracts from wild type and *pglB1*::*aphA* genetic backgrounds were mixed (labeled pglB1 + wt) to demonstrate intermediate mobility of HgpAhis from different mutants.

To identify genes involved in HgpA *N*-linked glycosylation, we constructed insertional knockout mutants in seven *H. pullorum pgl*/*wbp* genes from five loci (Figure 1). These mutants were constructed in the *H. pullorum* Hp47 genetic background that produces HgpAhis_10_ (see above and Table SIII) and the relative electrophoretic mobility of the *N*-linked glycoprotein investigated by SDS-PAGE of whole-cell lysates followed by Western blotting with an anti-His antiserum. HgpAhis_10_ electrophoretic mobility increased when derived from a *pglB1* insertional knockout mutant compared to that derived from the wild-type strain indicating modification via a *pglB1* dependent pathway (Figure 4). Similar analyses of *H. pullorum* strains with disrupted glycosyltransferase-encoding *pglC*, *pglA*, *pglH* and *pglJ* genes resulted in increased but varying mobility of the corresponding HgpAhis_10_ proteins in SDS-PAGE (Figure 4) indicating their involvement in assembly of the *N*-linked pentasaccharide. We also investigated *wbpO* and *wbpS* (Figure 1), products of which have significant levels of amino acid sequence identity (32% and 63% respectively) to *Pseudomonas aeruginosa* WbpO and WbpS involved in biosynthesis of 2-acetamido-2-deoxy-d-galacturonamide (GalNAcAN). In *P. aeruginosa*, WbpO converts UDP-GlcNAc to UDP-2-acetamido-2-deoxy-d-glucuronate (UDP-GlcNAcA), and the activated form of this sugar is then converted to UDP-GalNAcA by the isomerase WbpP (King et al. 2010). It was further proposed that WbpS amidotransferase activity is responsible for production of UDP-GalNAcAN (King et al. 2010). The role of *P. aeruginosa wbpOS* gene products in biosynthesis of these sugars indicated that the *H. pullorum* orthologues may be involved in biosynthesis of similar sugars and this is consistent with presence of HexNAcAN/HexNAcA in the *H. pullorum N*-linked pentasaccharide. Indeed insertional knockout mutagenesis of *wbpO* and *wbpS* resulted in increased mobility of the HgpAhis protein (Figure 4).

To further investigate HgpA produced in these *pgl/wbp* genetic backgrounds, a chromosomal *hgpA*his_6_ gene was introduced into these backgrounds (see Methods). The HgpAhis_6_ protein was purified and intact mass values were determined by electrospray ionization MS (Table II). The predicted mass of unmodified HgpAhis_6_ is 26,071 Da and the observed masses of HgpAhis_6_ proteins derived from the wild type and *pglB1*::*aphA* mutant were 27,127 and 26,070 Da, respectively. This difference in electrophoretic mobility and mass indicates *pglB1*-dependent modification of HgpAhis_6_ with a presumed *N*-linked glycan of 1056 Da, consistent with previous data (Jervis et al. 2010). The intact mass value of 26,069 Da for HgpAhis_6_ derived from the *H. pullorum pglC* insertional knockout mutant was also consistent with production of unmodified protein. Insertional knockout mutagenesis of four further *H. pullorum* genes (*pglA*, *pglJ*, *pglH* and *wbpS*) produced HgpAhis_6_ proteins of masses 26,297, 26,488, 26,923 and 26,490 Da, respectively (Table II). These values were intermediate between those obtained for HgpAhis_6_ from wild type and *pglB* or *pglC* insertional knockout mutants indicating modification with truncated glycans. The HgpAhis_6_ protein from the *wbpO* knockout mutant did not give consistent values in intact mass analysis experiments likely due to sample heterogeneity. The masses obtained for HgpAhis_6_ proteins derived from mutant strains were consistent with their varying electrophoretic mobility, and combined intact mass and western blotting data demonstrate that *H. pullorum pgl* and *wbp* gene products are involved in HgpAhis_6_ modification.

**Table II.** Intact mass values of HgpA determined by LC-ESI–MS in *H. pullorum pgl* and *wbp* gene insertional knockout mutants

*H. pullorum* genetic background	HgpAhis intact mass (Da)
NCTC 12824 23S::eryhgpAhis6 (Hp31)	27,127
NCTC 12824 23S::eryhgpAhis6 *pglB1*::*aphA* (Hp67)	26,070
NCTC 12824 23S::eryhgpAhis6 *pglA*::*aphA* (Hp25)	26,297
NCTC 12824 23S::eryhgpAhis6 *pglC*::*aphA* (Hp26)	26,069
NCTC 12824 23S::eryhgpAhis6 *pglH*::*aphA* (Hp27)	26,923
NCTC 12824 23S::eryhgpAhis6 *pglJ*::*aphA* (Hp29)	26,488
NCTC 12824 23S::eryhgpAhis6 *wbpS*::*aphA* (Hp68)	26,490

### Structural characterization of *N*-linked glycans produced in mutant backgrounds

**Fig. 5.**
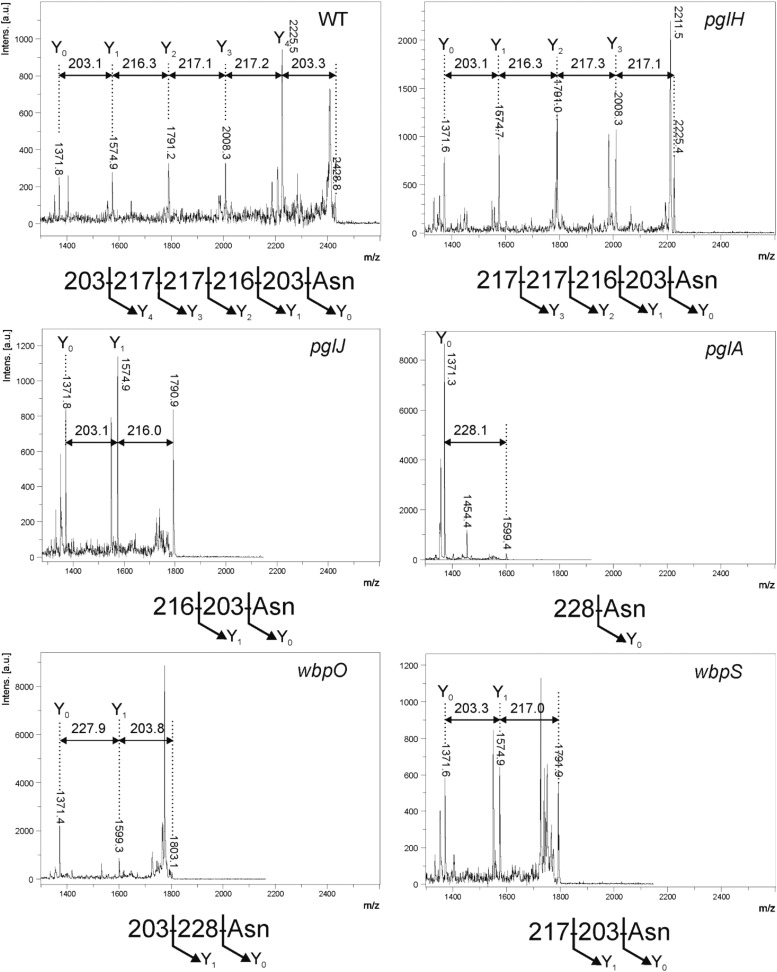
Tandem MALDI mass spectrometry of the HgpA tryptic (glyco)peptide derived from wild type *H. pullorum* and insertional knockout mutants of *pglHJA* and *wbpOS* genes. Spectra produced from fragmentation of the tryptic glycopeptide (EIPTPNENNDTK – 1371.4 Da) containing the *N*-glycosylation site of the glycoprotein HgpAhis with inferred structures of the corresponding *N*-linked glycans below. The genetic backgrounds from which HgpA protein was purified are indicated top right of each spectrum.

To further characterize the variety of HgpA modifications produced in *pgl/wbp* mutant backgrounds, purified HgpAhis_6_ proteins were digested with trypsin and peptides analyzed by MALDI-TOF MS. A number of peaks were observed corresponding to the predicted masses of HgpAhis-derived tryptic peptides confirming protein identity (data not shown). However, a peak at *m*/*z* 2429 was present in the spectrum for HgpAhis derived from the wild type *H. pullorum* but not from the *pglB1* knockout. This *m*/*z* value corresponds to the mass of the predicted tryptic peptide containing the *N*-linked glycosylation sequon (EIPTPNENNDTK – 1371.4 Da) plus the mass of the *H. pullorum* pentasaccharide *N*-linked glycan (1056 Da). This peak was selected for fragmentation by MALDI-LIFT-TOF/TOF MS and the resultant spectrum (Figure 5) is consistent with the previously observed HexNAc-216-217-217-HexNAc pentasaccharide observed on an in vitro generated glycopeptide (Jervis et al. 2010) and present on glycoproteins identified above. Insertional knockout mutagenesis of the *H. pullorum pglH* gene results in production of an HgpAhis-derived glycopeptide with a tetrasaccharide structure, lacking the nonreducing end residue (Figure 5). Similarly, inactivation of the *pglJ* gene resulted in production of an *N*-linked disaccharide glycan consisting of the first two sugars of the pentasaccharide (Figure 5). Inactivation of the *pglA* gene resulted in production of a monosaccharide *N*-linked glycan, although the spectrum produced indicated presence of a diNAcBac rather than a HexNAc residue (Figure 5). In a *pglC* insertional knockout mutant there was no detectable *N*-linked glycan suggesting its role as the initiating transferase as seen in *C. jejuni* (Linton et al. 2005; Glover et al. 2006). These data indicate that biosynthesis of the *H. pullorum N*-linked pentasaccharide glycan involves sequential action of the PglC, PglA, PglJ and PglH glycosyltransferases and are entirely consistent with the intact mass analysis for each of the mutants described above. The MALDI-LIFT-TOF/TOF MS spectra generated from HgpAhis produced in both the *wbpO* and *wbpS* mutants indicated presence of a disaccharide *N*-linked glycan. In the *wbpO* mutant the disaccharide consisted of a diNAcBac reducing end sugar and a HexNAc residue, whilst in the *wbpS* mutant the disaccharide was composed of a reducing end HexNAc and a HexNAcAN residue, again consistent with intact mass data presented above (Figure 5).

### Demonstration of further *H. pullorum* N-linked glycoproteins

**Fig. 6.**
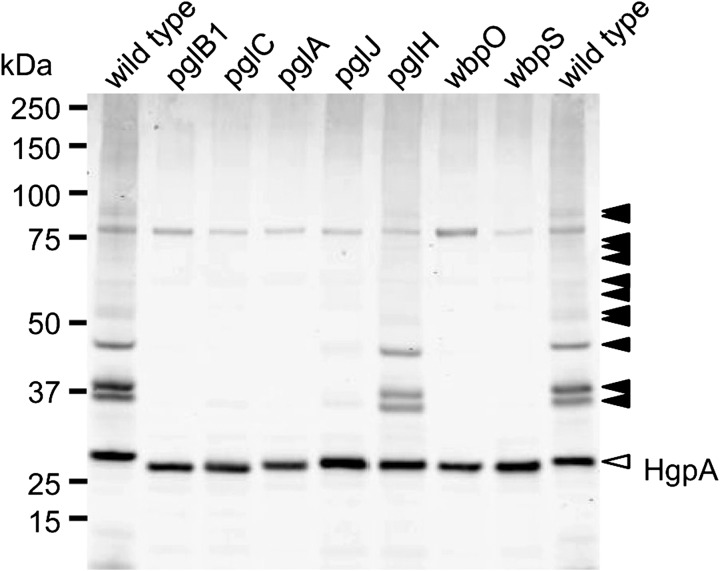
Immunoreactivity of whole-cell lysates from *H. pullorum pgl* and *wbp* gene mutants. Whole cell lysates from *H. pullorum* NCTC 12824 and corresponding *pglBCAJH* and *wbpOS* insertional knockout mutants were separated by SDS-PAGE, Western blotted and probed with a polyclonal antibody raised against purified HgpA glycoprotein. Shaded arrowheads indicate position of bands corresponding to putative glycoproteins whilst the unshaded arrowhead indicates the band corresponding to HgpA itself.

To identify further *N*-linked glycoproteins, a polyclonal antiserum was raised against purified HgpAhis_10_ from *H. pullorum* (see Methods). When a wild type *H. pullorum* whole-cell lysate was probed with this antiserum a number of immunoreactive bands were observed (Figure 6, arrowheads). Major bands at 35–40 kDa are consistent with the predicted masses of proteins Hp00296c and Hp00561c identified by MS/MS (Table I), with additional minor bands above 50 kDa and approximately 90 kDa consistent with the identification of Hp01062c and Hp00314c, respectively. The majority of these bands (Figure 6; shaded arrowheads) were no longer detected in a *pglB1* insertional knockout mutant indicating these represented glycoproteins glycosylated in a *pglB1*-dependent manner. Their immunoreactivity was also dependent on *pglABC* and *wbpOS* genes demonstrating their role in this general protein glycosylation pathway. In *pglJ* and particularly *pglH* knockout backgrounds, bands retained some immunoreactivity suggesting that the antiserum recognizes the shortened glycans likely present on proteins in these backgrounds. Immunoreactivity of an approximately 28 kDa band was unaffected by *pgl* and *wbp* gene mutations, however the mobility of this band was increased in these mutants (Figure 6). This band likely represents HgpA detected by antibodies against both the *N*-linked glycan and the protein itself and this is consistent with a predicted size of 26 kDa for glycosylated HgpA. The increased HgpA mobility in these backgrounds is presumably due to reduction in size of glycan structures, an interpretation consistent with data obtained above. These and previous data demonstrate the functioning of an *H. pullorum* PglB1-dependent general protein glycosylation system.

## Discussion

**Fig. 7.**
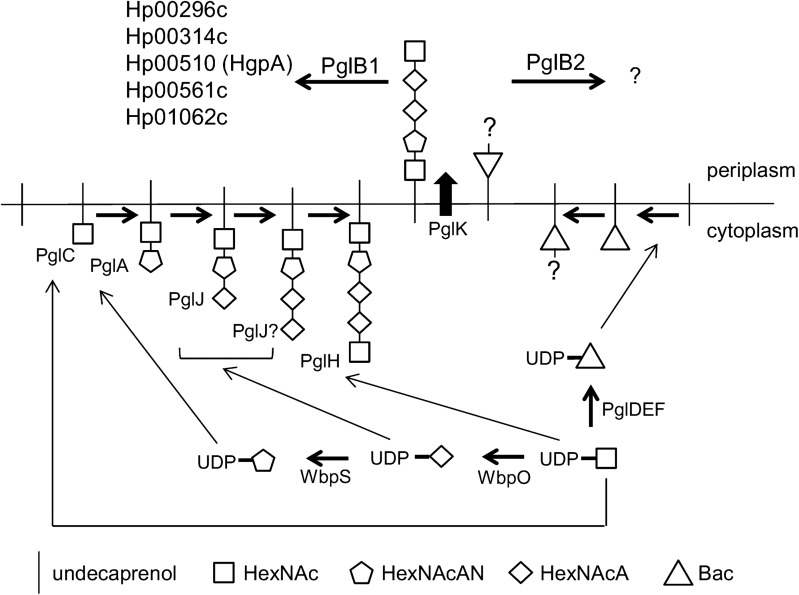
Model of *H. pullorum N*-linked protein glycosylation pathways. Direct evidence for role of PglCAJHB1 and WbpOS is provided in this study. The proposed roles of PglDEFK and PglB2 are based on established function of *C. jejuni* orthologues. The *H. pullorum* PglB1-dependent *N*-linked pentasaccharide glycan is assembled through sequential action of glycosyltransferases PglCAJH, flipped into the periplasm by PglK and transferred to proteins as indicated in the periplasm by PglB1. A second proposed glycan, with a reducing end 228 Da residue synthesized by PglDEF activity, is similarly assembled and flipped into the periplasm where it is transferred to as yet unidentified protein(s) via PglB2 activity.

It is well established that *C. jejuni* encodes a general *N*-linked protein glycosylation pathway and similar *N*-linked glycan structures have been identified in other *Campylobacter* species (Jervis et al. 2012; Nothaft et al. 2012). Less well characterized are the putative *N*-linked protein glycosylation systems present in a small number of species from the related *Helicobacter* genus. The Helicobacter *N*-linked protein glycosylation systems are notable for the presence of two distinct OTase encoding *pglB* genes (Figure 1), with the *H. pullorum* PglB1 protein more similar to the *C. jejuni* PglB (31% amino acid sequence identity) than to *H. pullorum* PglB2 (23% identity). Through a variety of approaches in *E. coli* (Jervis et al. 2010), *C. jejuni* and *H. pullorum* we have demonstrated *N*-linked oligosaccharyltransferase activity of *H. pullorum* PglB1 but not PglB2. Furthermore evidence was provided for a *H. pullorum* PglB1 directed general protein glycosylation pathway (Figure 6). MS/MS analysis of enriched glycopeptides confirmed the *H. pullorum N*-glycan structure and identified six sites of glycosylation within five proteins. Intriguingly, only five of six glycopeptides contained the anticipated *N*-linked bacterial sequon D/E-X-N-X-S/T, with a second glycopeptide in Hp00296c containing a nonclassical sequon with a lysine at the +2 position (EN**N**QK; Table I). Recent work demonstrated that *C. jejuni* PglB is able to glycosylate nonclassical sequons with three examples identified in the NCTC 11168O strain (Scott et al. 2014). These included sequons lacking the D/E at position −2, or S/T at position +2, but never both. A similar phenomenon has been described for *Campylobacter lari* PglB (Schwarz et al. 2011b). For Hp00296c, we noted that the occupied nonclassical sequon is close to the identified “classical” sequon (N171 versus N190; Table I), suggesting that hierarchical site occupancy as observed for the *C. jejuni* lipoprotein JlpA (Scott et al. 2009) may occur, however this is yet to be determined. Knockout of several genes from the *H. pullorum pgl* gene loci that encode putative glycosyltransferases and enzymes involved in sugar biosynthesis altered the structure of the PglB1-dependent *N*-linked glycan (Figures 4 and 5). Based on these data we propose a model for *H. pullorum N*-linked protein glycosylation (Figure 7). The proposed activities of *H. pullorum* PglCAJH are broadly consistent with those of their similarly named counterparts in *C. jejuni* (Glover et al. 2005a; Linton et al. 2005). The *H. pullorum wbpS* mutant produced an HgpA-linked disaccharide of HexNAc–HexNAcA (Figure 5). Again this is consistent with the predicted role for WbpS in the biosynthesis of UDP-HexNAcAN from UDP-HexNAcA in *P. aeruginosa* (King et al. 2010). Thus if the *H. pullorum wbpS* knockout mutant is unable to synthesize HexNAcAN, the structurally related and biosynthetic precursor HexNAcA is transferred to the reducing end HexNAc in its place with the PglJ transferase presumably unable to further extend the glycan structure. In the background of *pglA*::*aphA* and *wbpO*::*aphA* mutants, HgpA was modified with glycans including a reducing end diNAcBac residue absent in the pentasaccharide *N*-glycan produced in the wild type background (Figure 5), but consistent with the presence in *H. pullorum* of orthologues of the *C. jejuni pglDEF* genes encoding enzymes required for diNAcBac biosynthesis (Figure 1). Surprisingly the *H. pullorum pglDEF* genes are not required for biosynthesis of the PglB1-dependent *H. pullorum N*-linked pentasaccharide (Jervis et al. 2010) and we hypothesize that they are involved in biosynthesis of an as yet uncharacterized glycan potentially transferred by the *H. pullorum* PglB2 dependent pathway. Disruption of the PglB1 pathway at the earlier stages of pentasaccharide assembly may result in cross-talk between the two systems. Due to its similarity to *P. aeruginosa* WbpO it is plausible that *H. pullorum* WbpO converts UDP-HexNAc to UDP-HexNAcA. Indeed disruption of *wbpO* resulted in substitution of the HexNAcAN of the *N*-linked glycan with a HexNAc residue albeit with a diNAcBac reducing end. Despite this, we found no evidence of enriched glycopeptides with Asn-linked glycans containing a diNAcBac reducing end sugar in the *H. pullorum* wild-type strain. Glycopeptide data analysis was performed by manual interpretation of all MS/MS scans containing the HexNAc oxonium ion 204.086 *m*/*z*, which would identify glycopeptides with truncated HexNAc-containing glycans or those containing di-NAcBac–HexNAc disaccharides. Searches were also performed to extract MS/MS scans containing di-NAcBac-associated ions, however bacillosamine does not produce intense oxonium ions during vibrational fragmentation approaches. Database searches using diNAcBac, diNAcBac + HexNAc and other combinations of possible glycan masses also failed to identify additional glycopeptides (data not shown). This suggests that, in accordance with the production of diNAcBac glycopeptides in *pglA*/*wbpO* mutants, such glycans are only produced under specific conditions that influence *pglA*/*wbpO* expression and these are yet to be determined. We have shown the involvement of a number of *pgl* genes in the HpPglB1-dependent glycosylation system but further genes associated with these *pgl* loci remain uncharacterized. These include *pglL* and *pglI* encoding putative glycosyltransferases, which we were unable to disrupt and the putative di-NAcBac synthesis genes *pglDEF* that are not involved in the HpPglB1-dependent system (Jervis et al. 2010). This suggests the *N*-linked protein glycosylation system in *H. pullorum* may be more complex than in the *C. jejuni* model. The parasites *Leishmania major* and *Trypanosoma brucei* possess four and three single subunit *N*-linked OTases, respectively, with different glycan and acceptor protein specificities and varying growth phase dependent expression patterns resulting in production of distinct subsets of the *N*-linked glycoproteome (Nasab et al. 2008; Izquierdo et al. 2009). Archaeal species with multiple OTases have not yet been studied experimentally though *Haloferax volcanii* has been shown to produce two structurally distinct S-layer protein N-linked glycans (Kaminski et al. 2013a) and *Archaeoglobus fulgidus* encodes three OTases and produces two structurally distinct LLOs (Taguchi et al. 2016). Genetic analysis shows that most OTase gene duplication events are relatively ancient (Kaminski et al. 2013b). Our combined data enable proposal of a model for *N*-linked protein glycosylation in *H. pullorum* (Figure 7) with experimental evidence for biosynthesis of pentasaccharide transferred onto proteins in the periplasm by PglB1 and a more speculative proposal for PglB2 function that will require experimental verification.

## Materials and methods

### Bacterial strains

All *E. coli* strains were grown in Luria–Bertani (LB) broth or on LB agar plates. *C. jejuni* NCTC 11168 and *H. pullorum* NCTC 12824 strains were grown on Columbia agar containing 5% defibrinated horse blood (TCS Biosciences) at 42°C in a modified atmosphere (85% N_2_, 10% CO_2_ and 5% O_2_) generated with a VA500 workstation (Don Whitley Ltd.). Antibiotics were used at the following concentrations: kanamycin 50 μg/mL, chloramphenicol 17 μg/mL, tetracycline 10 μg/mL, ampicillin 100 μg/mL and erythromycin 300 μg/mL. Primers, plasmids and strains are described in Tables S1, S2 and S3, respectively.

### Reverse transcriptase PCR

Total RNA was extracted from *H. pullorum* harvested from 48 h blood agar plates using the Qiagen RNeasy kit with an additional in-solution DNAse I digestion step. Reverse transcriptase PCR was performed using the Qiagen OneStep RT PCR kit according to manufacturer’s instructions.

### Integration of pglB genes onto the *C. jejuni* chromosome

Various *pglB* genes were integrated onto the *C. jejuni* 11168 *pglB*::*aphA* chromosome using a modification of a previous method (Gerber et al. 2013). A chloramphenicol resistance cassette (van Vliet et al. 1998) was PCR amplified using primers Cm-F (restriction sites SpeI, BglII and XhoI) and Cm-R (NcoI, NheI and SpeI), digested with SpeI and cloned into the SpeI site of a previously constructed vector consisting of pUC18 backbone with a region of the *C. jejuni* 11168 pseudogene Cj0223 cloned into the SmaI site (Hitchen et al. 2010). The resultant plasmid was named pCJC1.

Complete *C. jejuni pglB*, *H. pullorum pglB1* and *H. pullorum pglB2* genes were PCR amplified with primer pairs PglBCjcomp-F/PglBCjcomp-R, HppglB1comp-F/HppglB1comp-R and HppglB2comp-F/HppglB2comp-R, respectively, to include approximately 70 bp upstream of the each start codon. Primers PglBCjcomp-F, HppglB1comp-F and HppglB2comp-F included an NcoI site at the 5’ end and the corresponding reverse primers encoded a deca-histidine tag at the 3’ end along with either an NheI (PglBCjcomp-R) or SpeI (HppglB1comp-R and HppglB2comp-R) site. Products were digested with NcoI and either NheI or SpeI as appropriate and cloned immediately downstream of, and in the same transcriptional orientation as, the chloramphenicol resistance cassette of pCJC1. The resulting plasmids pCJC1pglBCj, pCJC1pglHp1 and pCJC1HppglB2 (Table SII) were electroporated into *C. jejuni* 11168 *pglB*::*aphA* cells, chloramphenicol-resistant colonies selected and the anticipated double crossover integration events verified by PCR.

### Expression of hgpA in *E. coli*

The predicted *H. pullorum* NCTC 12824 *hgpA* coding sequence was PCR amplified with primers hgpA-F and hgpA-R to introduce SphI and BglII restriction sites at the 5’ and 3’ end, respectively. Following digestion with SphI and BglII, PCR products were ligated into similarly digested vector pQE70 to generate pQEhgpA encoding a C-terminal hexa-his tagged protein. A variant, pQEhgpAN46Q, was created by site directed mutagenesis using primers hgpAN46SDM-F and hgpAN46SDM-R. Plasmids pQEhgpA and pQEhgpAN46Q were transformed into *E. coli* Novablue (Stratagene) cells harboring pACYCpglB::aphA (24) along with plasmids expressing either *C. jejuni pglB* (pMAF10), *H. pullorum pglB1* (pMLHp1) or *H. pullorum pglB2* (pMLHp2) (Jervis et al. 2010). Transformants were grown overnight at 37°C, diluted 1 in 100 in LB broth supplemented with appropriate antibiotics, grown to an optical density at 600 nm (A_600_) of 0.6 and expression of *hgpA*his and *pglB* genes induced with 1 mM IPTG and 0.2% (w/v) arabinose. Following overnight incubation at 37°C cultures were harvested, normalized by A_600_, resuspended in SDS-PAGE loading buffer and incubated at 95°C for 5 min. Whole-cell lysates were separated by SDS-PAGE, transferred to nitrocellulose membrane, and probed with hR6 antiserum (gift from Markus Aebi) followed by an IRDye 800CW goat anti-rabbit IgG secondary antibody (LI-COR) and mouse anti-penta-His (Qiagen) followed by goat anti-mouse IgG secondary antibody (LI-COR). Blots were imaged using a LI-COR Odyssey Infrared Imaging System.

### Introduction of oligo-histidine tagged hgpA onto the *H. pullorum* chromosome

A 1.8 kb internal fragment of the *H. pullorum* 23S rRNA gene (*rrl*) was amplified using primers Hp23S-F and Hp23S-R, ligated into pGEM T-easy to create plasmid pHPC and digested with HindIII to excise 276 bp of *rrl*. An erythromycin resistance cassette (*ermC*) lacking a transcriptional terminator was amplified using primers Ery-F (containing a HindIII site) and Ery-R (HindIII and BamHI sites), and ligated into HindIII digested pHPC in the same transcriptional orientation as flanking *rrl* fragments to create (pHPC1). A derivative of this plasmid, termed pHPC2, was constructed by cloning the promoter region of the *C. jejuni porA* gene into the pHPC1 BamHI site located immediately downstream of the erythromycin resistance cassette with the promoter in the same transcriptional orientation as *rrl* gene and erythromycin cassette. The promoter region was PCR amplified using primers porAP-F (containing XhoI site) and porAP-R (NdeI and XhoI sites). Two *H. pullorum hgpA* expression systems were constructed based on either pHPC1 or pHPC2. In the first the *hgpA* ORF was PCR amplified using primers hgpABamHI-F and hgpABamHI-R to incorporate a C-terminal hexa-his tag and ligated into the BamHI site at the 3’ end of the erythromycin resistance cassette in pHPC1 to create pHPC1hgpAhis. In the second, the *hgpA* ORF was PCR amplified with primers hgpANdeI-F and hgpANdeI-R to include a C-terminal deca-his tag and cloned into the NdeI site of the plasmid pHPC2 downstream of the *porA* promoter region to create plasmid pHPC2hgpAhis.

Plasmids pHPC1hgpAhis and pHPC2hgpAhis were electroporated into *H. pullorum* cells (van Vliet et al. 1998; Jervis et al. 2010) and erythromycin resistant colonies screened by PCR for the predicted double crossover events within the chromosomal *rrl* gene.

### Purification of HgpAhis from *H. pullorum*

Approximately 1 g of *H.* pullorum NCTC 12824 *rrl*::*ermC hgpA*his_6_ (Hp31) cells were resuspended in 3 mL of Binding Buffer (50 mM Na_2_HPO_4_, 300 mM NaCl, 30 mM imidazole, pH 8.0) containing protease inhibitors phenylmethanesulfonylfluoride (0.1 mM) and benzamidine (1 mM). Cells were lysed in a French press (Thermo Scientific, UK), centrifuged at 8000 × *g* for 20 min and the supernatant incubated with 150 μL of Ni-NTA Magnetic Agarose Beads (Qiagen, UK) for 1 h at room temperature with mixing. Beads were washed three times with 1 mL of Binding Buffer and bound protein eluted in 50 μL of 50 mM Na_2_HPO_4_, 300 mM NaCl, 500 mM imidazole, pH 8.0 at room temperature for 5 min.

### Intact mass analysis by ESI–MS

Purified proteins were dialyzed against 25 mM Tris-HCl, 25 mM NaCl (pH 8.0) and analyzed by LC-ESI–MS using a Dionex PepSwift RP column (200 μm × 50 mm) connected to a Micromass LCT ESI–MS. Spectra were deconvoluted using the MaxEnt I software (Micromass).

### Glycan analysis by MALDI-MS

Coomassie stained SDS-PAGE bands were excised, lyophilized and digested with trypsin (E.C.3.4.21.4, Promega) overnight. Peptides were extracted from gel pieces using a C18 ZipTip (Millipore, UK) according to the manufacturer’s protocol and eluted in 10 μL of 50% acetonitrile, 0.1% formic acid. MALDI-TOF MS and MALDI-LIFT-TOF/TOF MS spectra were acquired by laser-induced dissociation (LID) using a Bruker Ultraflex II mass spectrometer in the positive-ion reflection mode with a matrix of 20 mg/mL 2,5-dihydroxybenzoic acid (DHB) (30% acetonitrile, 0.1% TFA). Data were analysed with FlexAnalysis 3.0 software (Bruker Daltonics).

### Enrichment of glycopeptides using zwitterionic-hydrophilic interaction liquid chromatography (ZIC-HILIC) and identification by reversed phase LC–MS/MS

Identification of glycopeptides from *H. pullorum* NCTC 12824 WT was conducted as previously described (17, 33). Lysates were suspended in 6 M urea, 2 M thiourea, 40 mM NH_4_HCO_3_, and reduced and alkylated with 20 mM dithiothreitol and 40 mM iodoacetamide, respectively, each for 1 h at room temperature. Samples were diluted 1:10 with 40 mM NH_4_HCO_3_ and digested with porcine sequencing grade trypsin (Promega, Madison WI; 1:100) overnight at 37°C. Peptides were acidified with 2% (v/v) formic acid and 0.1% (v/v) TFA, then desalted by hydrophilic lipophilic-balance solid phase extraction (HLB-SPE) (Waters, Milford MA). ZIC-HILIC enrichments were carried out according to Scott et al. (2011). Fractions were resuspended in 0.1% formic acid and loaded directly onto a 20 cm, 75 μm inner diameter, 360 μm outer diameter Reprosil Gold C_18_ AQ 1.9 μm (Dr. Maisch, Ammerbuch-Entringen, Germany) reversed phase (RP) column using a trapless EASY-nLC II system (Proxeon, Odense Denmark) coupled to an LTQ-Orbitrap Velos Pro mass spectrometer (Thermo Scientific, San Jose, CA). Peptides were loaded in 95% buffer A (0.1% FA) and eluted at 250 nL/min using a linear gradient of buffer B (80% ACN, 0.1% FA) from 5% to 40% over 120 min. The column was washed with 90% buffer B for 10 min before being returned to 95% buffer A. The LTQ-Orbitrap Velos Pro was operated using Xcalibur v2.2 (Thermo Scientific) with a capillary temperature of 200°C in a data-dependent mode automatically switching between MS and higher energy collisional dissociation (HCD)/collision-induced dissociation (CID) MS/MS. For each MS scan, the three most abundant precursor ions were selected for HCD (normalized collision energy 45) and CID (normalized collision energy 35). Data processing was carried out as previously described (Scott et al. 2014). Briefly, HCD scans from.raw files were processed in Proteome Discoverer v1.4.1.14 (Thermo Scientific) and searched using SEQUEST against an in-house, translated *H. pullorum* NCTC 12824 database. MS/MS scans that did not result in identifications were exported as.mgf files. The “mgf graph” feature within the MSMS module of GPMAW 10.0 (Lighthouse Data, Odense, Denmark) was used to highlight all scan events containing the diagnostic HexNAc oxonium ion 204.086 *m*/*z*, in addition to the oxonium ions for the HexNAcA (218.0665 *m*/*z*) and HexNAcAN (217.0824 *m*/*z*) species that are constituents of the *H. pullorum N*-glycan. MASCOT v2.2 searches were conducted against the *H. pullorum* NCTC 12824 database with parent ion mass accuracy of 20 ppm and product ion accuracy of 0.02 Da, no protease specificity, instrument set to MALDI-QIT-TOF, as well as the fixed modification carbamidomethyl (C) and variable modifications oxidation (M) and deamidation (N). All spectra were searched with the decoy option enabled, and no matches were detected (FDR 0%). HCD and CID scans from matched spectra (MASCOT scores >20) were manually inspected to ensure all major peaks were matched, and to validate attachment and composition of the *N*-glycan. Isotopic distribution analysis was performed with the MS Isotope module of Protein Prospector (http://prospector.ucsf.edu/prospector).

### Construction of *H. pullorum* insertional knockout mutants

*H. pullorum* genes were inactivated by insertion of the *aphA* gene via double crossover recombination events with appropriately constructed suicide vectors introduced into cells by electroporation (van Vliet et al. 1998). To create suicide vectors, PCR products of approximately 2 kbp were generated that incorporated regions of target genes. These were ligated into pGEM-T Easy, and the *aphA* cassette lacking a transcriptional terminator cloned into BamHI or HindIII sites within the central region of cloned PCR products. If these restriction sites were not present they were introduced by site directed mutagenesis or overlap PCR as described previously (Jervis et al. 2010). The individual mutations were made as described below.

*pglA*: Primers pglA-F and pglA-R were used to amplify a 1.6 kb fragment encompassing the complete *pglA* gene with a naturally occurring central HindIII restriction site.
*pglC*: A 729 bp fragment consisting of the first 111 bp at the 5′ end of *pglC* plus upstream region was amplified using primers pglC-UF and pglC-UR to include a 3′ HindIII site. A 689 bp fragment consisting of 340 bp of the 3′ end of *pglC* and downstream region was amplified using primers pglC-DF and pglC-DR to include a 5′ HindIII site. Overlap PCR was performed using primers pglC-UF and pglC-DR.
*pglH*: A 912 bp fragment consisting of the first 561 bp at the 5′end of *pglH* plus upstream region was amplified using primers pglH-UF and pglH-UR to include a 3′ HindIII site. A 929 bp fragment consisting of 527 bp of the 3′ end of *pglH* and downstream region was amplified using primers pglH-DF and pglH-DR to include a 5′ HindIII site. Overlap PCR was performed using primers pglH-UF and pglH-DR.
*pglI*: An 823 bp fragment consisting of the first 236 bp at the 5′end of pglI plus upstream region was amplified using primers pglI-UF and pglI-UR to include a 3′ HindIII site. An 857 bp fragment consisting of 651 bp of the 3′ end of *pglI* and downstream region was amplified using primers pglI-DF and pglI-DR to include a 5′ HindIII site. Overlap PCR was performed using primers pglI-UF and pglI-DR.
*pglJ*: An 879 bp fragment consisting of the first 204 bp at the 5′end of *pglJ* plus upstream region was amplified using primers pglJ-UF and pglJ-UR to include a 3′ HindIII site. An 808 bp fragment consisting of 196 bp of the 3′ end of *pglJ* and downstream region was amplified using primers pglJ-DF and pglJ-DR to include a 5′ HindIII site. Overlap PCR was performed using primers pglJ-UF and pglJ-DR.
*pglL*: An 857 bp fragment consisting of the first 180 bp at the 5′end of *pglL* plus upstream region was amplified using primers pglL-UF and pglL-UR to include a 3′ HindIII site. An 885 bp fragment at the 3′ end of *pglL* was amplified using primers pglLD-F and pglLD-R to include a 5′ HindIII site. Overlap PCR was performed using primers pglLU-F and pglLD-R.
*wbpO*: Primers wbpO-F and wbpO-R were used to amplify a 1.9 kb fragment including the complete *wbpO* CDS with a naturally occurring central HindIII restriction site.
*wbpS*: Primers wbpS-F and wbpS-R were used to amplify a 1.2 kb internal fragment of *wbpS*. A BamHI restriction site was created using site directed mutagenesis with primers wbpSBamHI-F and wbpSBamHI-R.

## Supplementary Material

Supplementary Data
