# Supplementary material for: Functional analysis of the Helicobacter pullorum N-linked protein glycosylation system
Source: Glycobiology. 2018 Jan 11;28(4):233–44. doi: 10.1093/glycob/cwx110 (PMC6025236; doi:10.1093/glycob/cwx110)
Supplement: Supplementary Data [file supplementary_data.pdf]

```
H. pullorum2      MGKEKQEEAHNSQKDNQFPSTCLDSCFIQKSLKISLFFKPLFLWHFIFICIFCCAYFLFH        60
H. pullorum1      ---------------------------MQNKNW---LLIAVFVYLI---AILLRF-----        22
C. lari           --------------------MKLQQNFTDNNSIKYTCILILIAFAF---SVLCRL-----        32
C. jejuni         ---------------------MLKKEYLKNP-YLVLFAMIVLAYVF---SVFCRF-----        30
                                           .:          :: : :   .::

H. pullorum2      YWDYLLFLKDSENFFNHSLILTSYDSYFYAKGAKEFLESLNVAVP---YLSILAGVFAKI        117
H. pullorum1      YYPFV-LGEFEEYFYHNALLLNTNDGYFYAQGARDILAGVKSTF-YSPTHEILSQ-----        75
C. lari           YWVAW-ASEFYEFFFNDQLMITTNDGYAFAEGARDMIAGFHQPNDLSYFGSSLST-----        86
C. jejuni         YWVWW-ASEFNEYFFNNQLMIISNDGYAFAEGARDMIAGFHQPNDLSYYGSSLST-----        84
                  *:      :  * *:.. *:: : *.* :*:**:::: ...         . *:

H. pullorum2      FGLDNVLVWSSVAFSVSFG-IVLYGICFF----VLEYFKILTSKSNQIFAFLGAFLGAFA        172
H. pullorum1      -----ISAFLALILPFSLEQIIFYMPGFFGSLIVFFVVFVSRDFGG-IPSFLCGILSAIS        129
C. lari           -----LTYWLYSILPFSFESIILYMSAFFASLIVVPIILIAREYKLTTYGFIAALLGSIA        141
C. jejuni         -----LTYWLYKITPFSFESIILYMSTFLSSLVVIPIILLANEYKRPLMGFVAALLASVA        139
                       :  :      .*:  *::*   *:    *.  . :  .      .*: .:*.:.:

H. pullorum2      PHFYQRTGAGYFDTDMLLLSLPLLTIFCLWLYIIKQKFHWLVLFGFLGFLSVNWHNGIQN        232
H. pullorum1      VSYYNRTMFGYYDTDMLVIVLAFGVGVVIVELMRKISFFGLVLLILLSGFGLVYYPSMRY        189
C. lari           NSYYNRTMSGYYDTDMLVLVLPMLILLTFIRLTINKDIFTLLLSPVFIMIYLWWYPSSYS        201
C. jejuni         NSYYNRTMSGYYDTDMLVIVLPMFILFFMVRMILKKDFFSLIALPLFIGIYLWWYPSSYT        199
                    :*:**  **:*****:: * :   . :     : .:. *:   .:  : : :: .

H. pullorum2      ILLAGFLLYLGYEGLFFCV-------KRYFKTPILEISSVFLIVLAPSNLGILF------        279
H. pullorum1      I-------FVGYVGILTIFGI-FDRNIRMVNGCLLSVLLGF-LLFSTHFVFWLFLCFCGV        240
C. lari           L-------NFAMIGLFGLYTLVFHRKEKIFY---LTIALM---IIALSMLAWQYKLALIV        248
C. jejuni         L-------NVALIGLFLIYTLIFHRKEKIFY---IAVILS---SLTLSNIAWFYQSAIIV        246
                  :        ..   *::           : .    : :       ::   :   :

H. pullorum2      LVFALLMIKTRK---MMIFCFLIACAY--AYFFGLFNPLIAQIKAYLFGEIQYS----SA        330
H. pullorum1      MIKFIQRLDRRIFDFVYCLLLVIFCG----ILAFRVVPEIFAS---VYVAQDSK--EIVG        291
C. lari           LLFAIFAFKEEKINFYMIW-ALIFISILILHLSGGLDPVLYQLKFYVFKASDVQNLKDAA        307
C. jejuni         ILFALFALEQKRLNFMIIG-ILGSATLIFLILSGGVDPILYQLKFYIFRSDESANLT-QG        304
                  ::  :  :. .          :         :   . * :      ::   :       .

H. pullorum2      YIYASVVDSILETSSHGLGILVQR-SGGWLLFVLGLIGFLCFGFYFVLKRHNFIYLCIFL        389
H. pullorum1      FGYASVMGTISEVSKIDFWNFVYRISGNLLWFVLGSVGIIL-----LFLKKREF---WIF        343
C. lari           FMYFNVNETIMEVNTIDPEVFMQRISSSVLVFILSFIGFIL-----LCKDHKSM---LLA        359
C. jejuni         FMYFNVNQTIQEVENVDFSEFMRRISGSEIVFLFSLFGFVW-----LLRKHKSM---IMA        356
                  : * .*  :* *...     :: * *.  : *::. .*::      :   :. :    :

H. pullorum2      FPFLLLGFASLELGVRFSLFLAPILAFGVVLFFAGILDIMRRFLKTSM-------VVF--        440
H. pullorum1      LPFLIMGFFGYFGGLRFTFYAVVVYALGIGYLLCFLLDIFKRKIKWA-----VIFGVVFV        398
C. lari           LPMLALGFMALRAGLRFTIYAVPVMALGFGYFLYAFFNFLEKKQIKLSLRNKNILLILIA        419
C. jejuni         LPILVLGFLALKGGLRFTIYSVPVMALGFGFLLSEFKAILVKKYSQL---TSNVCIVFAT        413
                  :*:* :** .   *:**::: . : *:*.  ::  :  :: :              :.

H. pullorum2      AGYVALFLATLEYSIPKPILTNQEIRSLQSFC--FLKDDIVFSWWDYGYALEYFTKAEVL        498
H. pullorum1      CGSVFPHLWHIKNYIIPPILENSEAKALQEIP--AKYGDYALAWWDYGYFVRYFARLNTF        456
C. lari           FFSISPALMHIYYYKSSTVFTSYEASILNDLKNKAQREDYVVAWWDYGYPIRYYSDVKTL        479
C. jejuni         ILTLAPVFIHIYNYKAPTVFSQNEASLLNQLKNIANREDYVVTWWDYGYPVRYYSDVKTL        473
                     :   :  :       :: . *   *:.:       * ..:****** :.*:: :.:

H. pullorum2      LDGGLHSGSINYPIAEILMNKSPILARNFSLILAQKMQNTPKN-Q-----WKLLFEQIIQ        552
H. pullorum1      VDGGIHSGKQNYPISFVLSAKNQKQSYNMAKLTFNHIEFEDFAKSH------------Q        503
C. lari           IDGGKHLGKDNFFSSFVLSKEQI-PAANMARLSVEYTEKSFKENYP-----DVLKAMVKD        533
C. jejuni         VDGGKHLGKDNFFPSFSLSKDEQ-AAANMARLSVEYTEKSFYAPQNDILKSDILQAMMKD        532
                  :*** * *. *:  :  *  ..   : *:: :  :  :                     :

H. pullorum2      ENKSNPNIFLDSLQKKDYNIGDLPKGEVYWVLPKRIMPLVANIHSFRNINLQNGKRLRES        612
H. pullorum1      YSQSKA---LE-ALKGDIDCVGKRSEDLYIILPLRMVRIFSTIMQFSQPKGE---INQGL        556
C. lari           YNKTSAKDFLESLNDKDFKFDTNKTRDVYIYMPYRMLRIMPVVAQFANTNPDNGEQEKSL        593
C. jejuni         YNQSNVDLFLASLSKPDFKIDTPKTRDIYLYMPARMSLIFSTVASFSFINLDTGVLDKPF        592
                   .::.    *    . * .     . ::*  :* *:  :.  : .*   : :     :

H. pullorum2      VFVYGD-MPLKSAEEYFVFSD-FYIK-------RSQDGIPL-------------------        644
H. pullorum1      LMVSK-----TKSKDKIIFQDNVFVDIKKGKVGILNQEISLNIAKMIDLKDS----SKSL        607
C. lari           FFSQANAIAQDKTTGSVMLDNGVEIINDFRALKVEGASIPL--KAFVDIESITNGKFYYN        651
C. jejuni         TFSTAY--PLDVKNGEIYLSNGVVLSDDFRSFKIGDNVVSV--NSIVEINSIKQGEYKIT        648
                   :              . :.: . :             : :

H. pullorum2      --------VKMFW------EGREIVMDFEYLKSNLVQWLIFRNNP--AMNLVFENDFVVV        688
H. pullorum1      EFDKNSSLVVLML-----GGGDYILCDKEYLETFYFRGMFLDNLDSNLFEKVLKNEKIAI        662
C. lari           EIDSKAQIYLLFLRE----YKSFVILDESLYNSSYIQMFLLNQYDQDLFEQITNDTRAKI        707
C. jejuni         PIDDKAQFYIFYLKDSAIPYAQFILMDKTMFNSAYVQMFFLGNYDKNLFDLVINSRDAKV        708
                            :            :: *    ::   .: ::: :     :: : :.    :

H. pullorum2      YQVRK   693
H. pullorum1      YKLKQ   667
C. lari           YRLKR   712
C. jejuni         FKLKI   713
                  ::::
```

Fig. S1. ClustalQ alignment of PglB sequences from *Campylobacter jejuni*, *Campylobacter lari* and *H. pullorum*. Two putative PglB enzymes are encoded by *H. pullorum* and both possess conserved residues shown as required for oligosaccharyl transferase activity (bold).

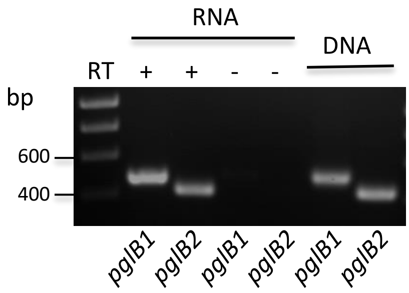

Fig. S2. Expression of *H. pullorum pglb1* and *pglB2.* RT-PCR analysis of total RNA extracted from *H. pullorum* NCTC 12824 indicates that both *pglB1*- and *pglB2*-specific transcripts are expressed during *in vitro* growth. RT indicates whether reverse transcriptase was added to the reaction, no product was detected in its absence. DNA template PCR controls indicated in lanes 7 and 8.

Precursor charge:	3	D R V D N G N G D V N G S K
Precursor MH+ (Da):	2503.92396	
Peptide mass (MH+):	1447.6175	

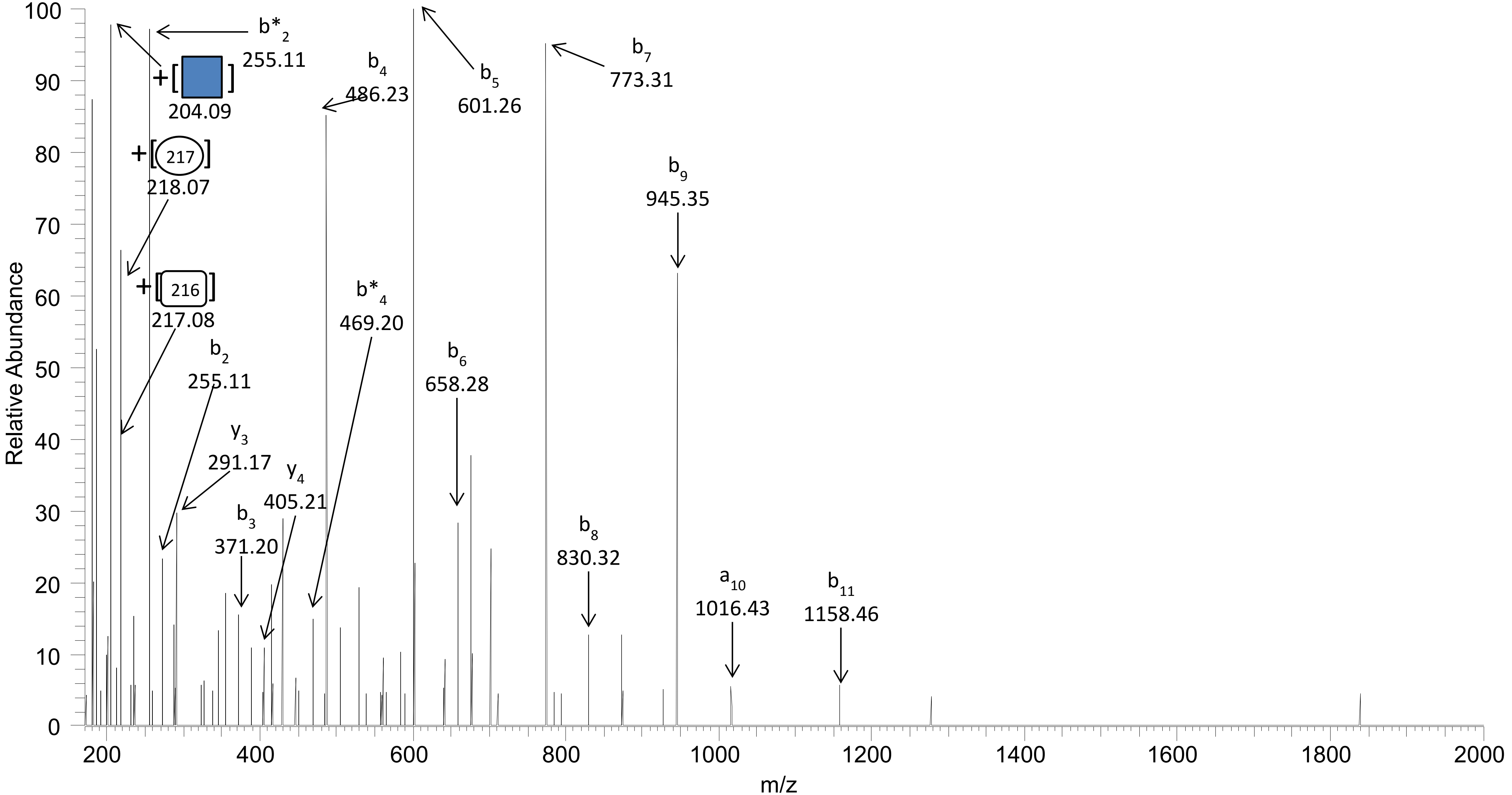

Precursor charge:	3
Precursor MH+ (Da):	2503.92396
Peptide mass (MH+):	1447.6175

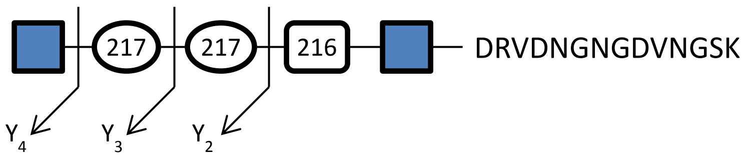

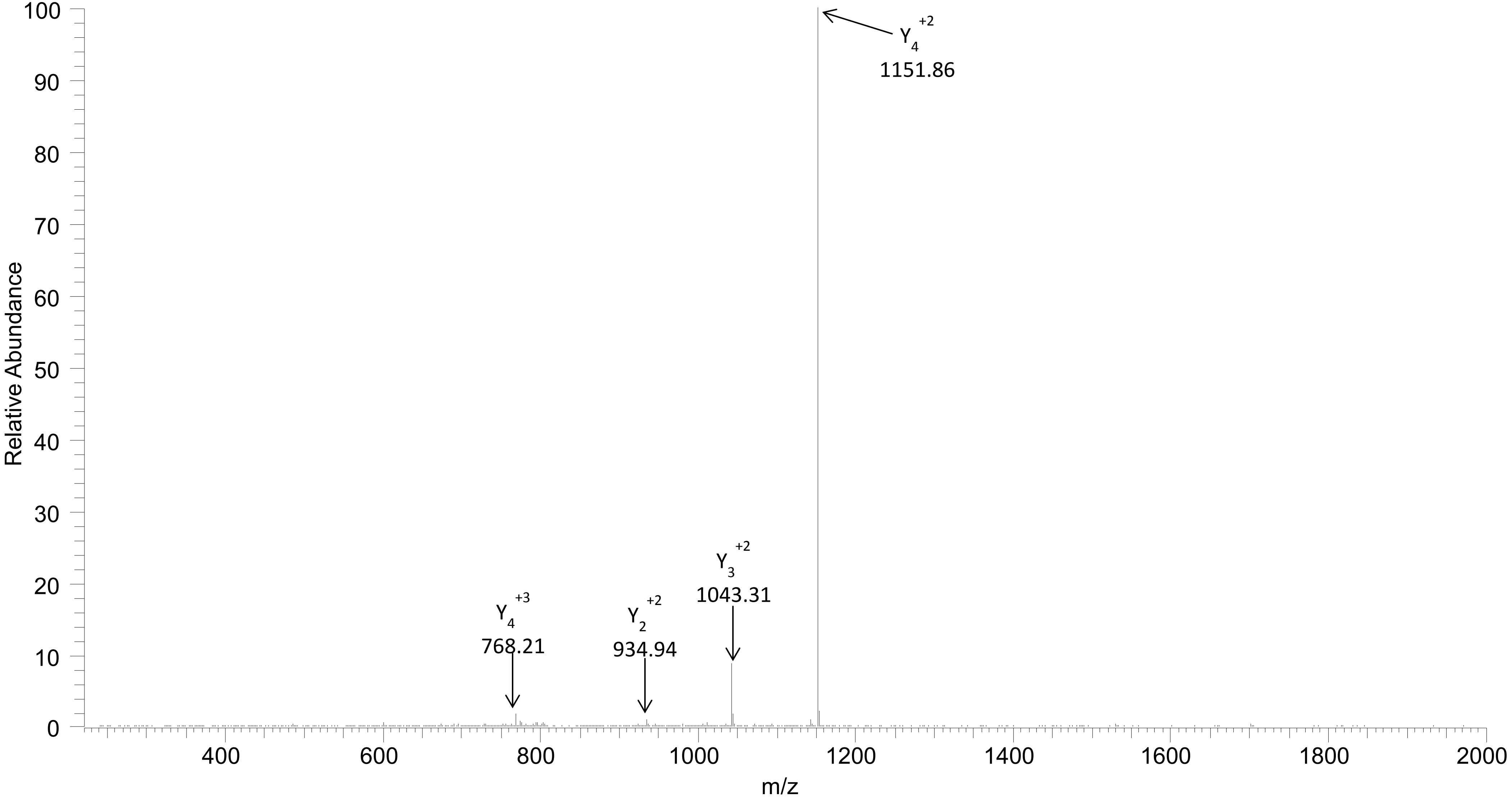

Precursor charge:	3	K D T I K E D S V E N N G S A P N A (b2 b3 b4 b5 b6 b7 b8 b9 b10 b11; y3)
Precursor MH+ (Da):	2945.17146	
Peptide mass (MH+):	1888.865	

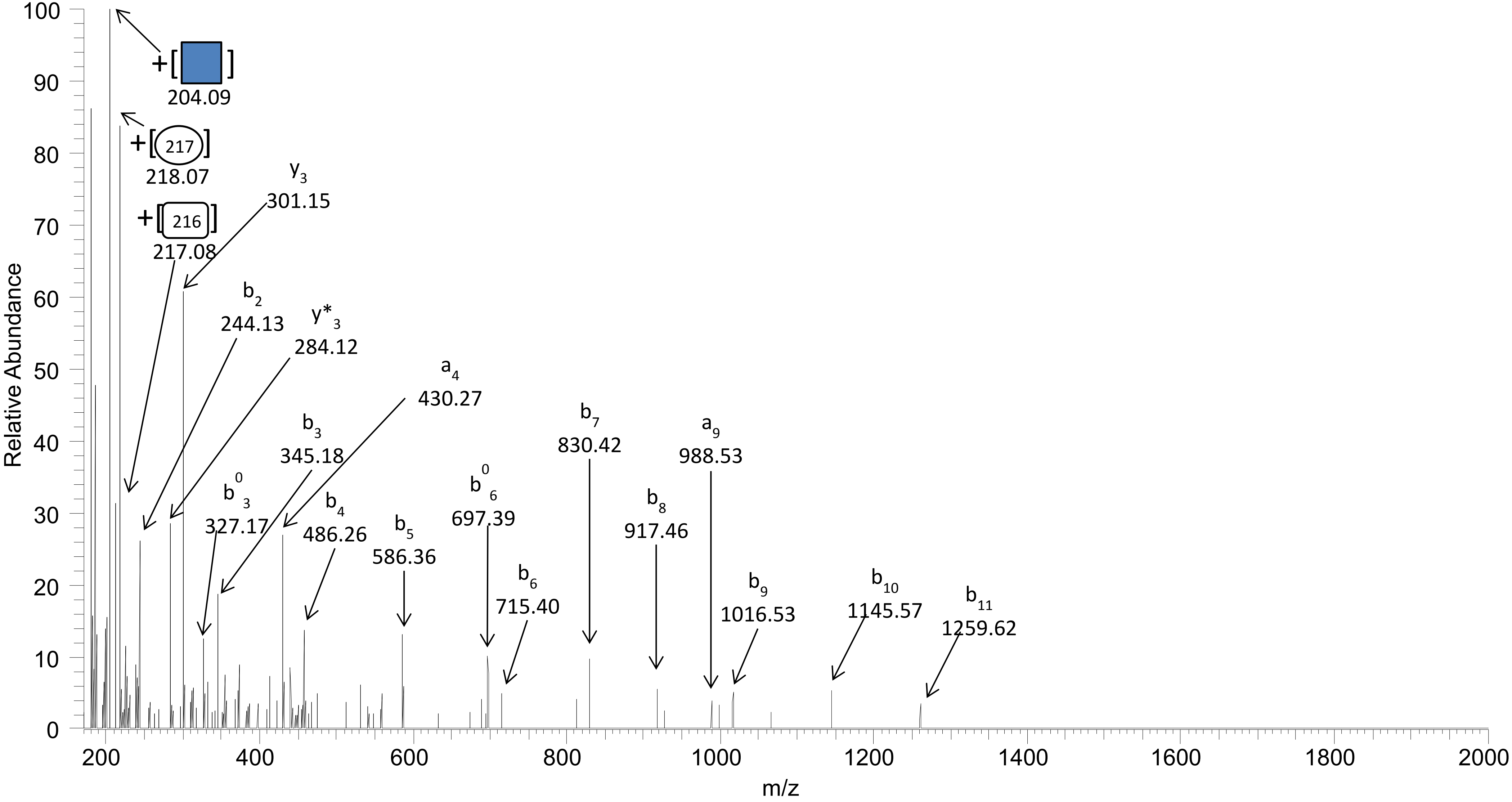

Precursor charge:	3
Precursor MH+ (Da):	2945.17146
Peptide mass (MH+):	1888.865

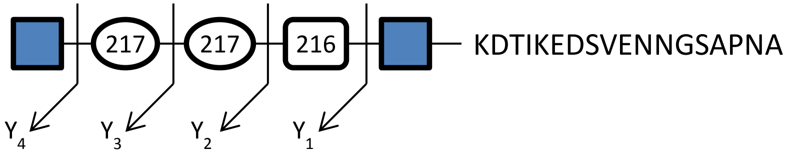

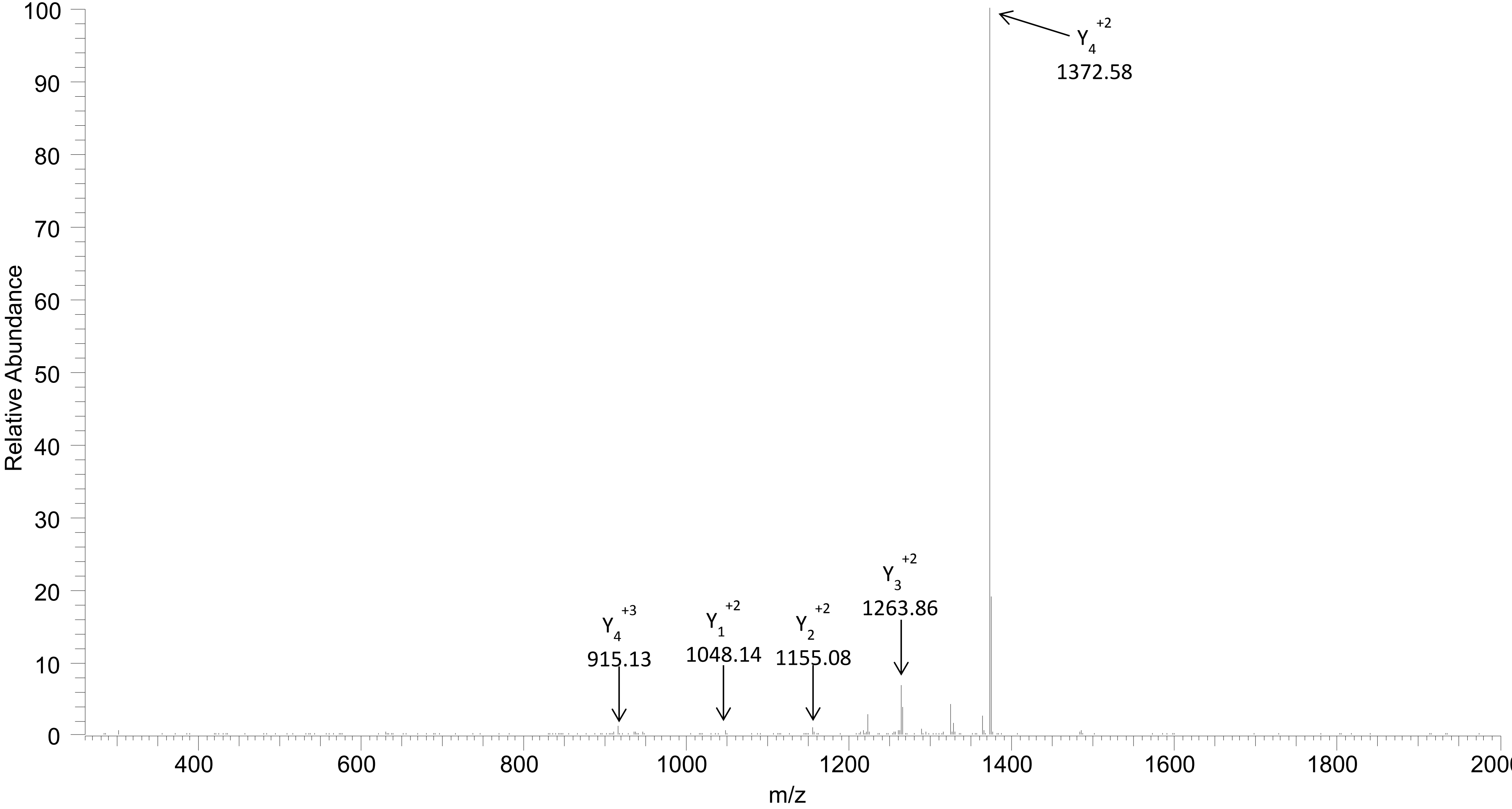

Precursor charge:	3	b2 b3 b4 b5 b6<br>P N A N A N I A T I E S A E N N Q K E S K Q
Precursor MH+ (Da):	3427.43156	
Peptide mass (MH+):	2371.1251	

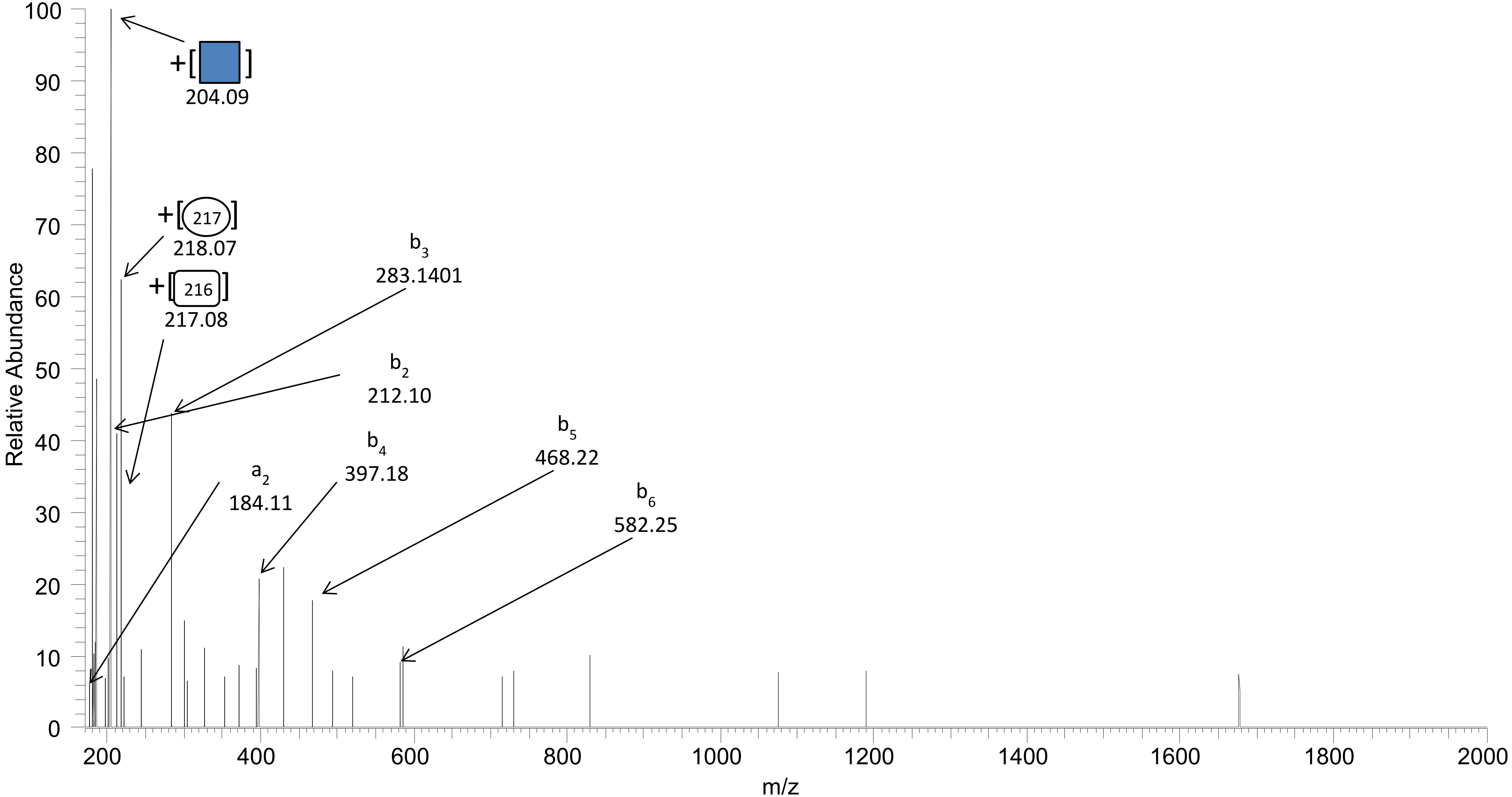

Precursor charge:	3
Precursor MH+ (Da):	3427.43156
Peptide mass (MH+):	2371.1251

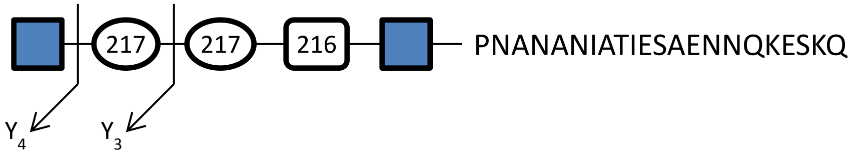

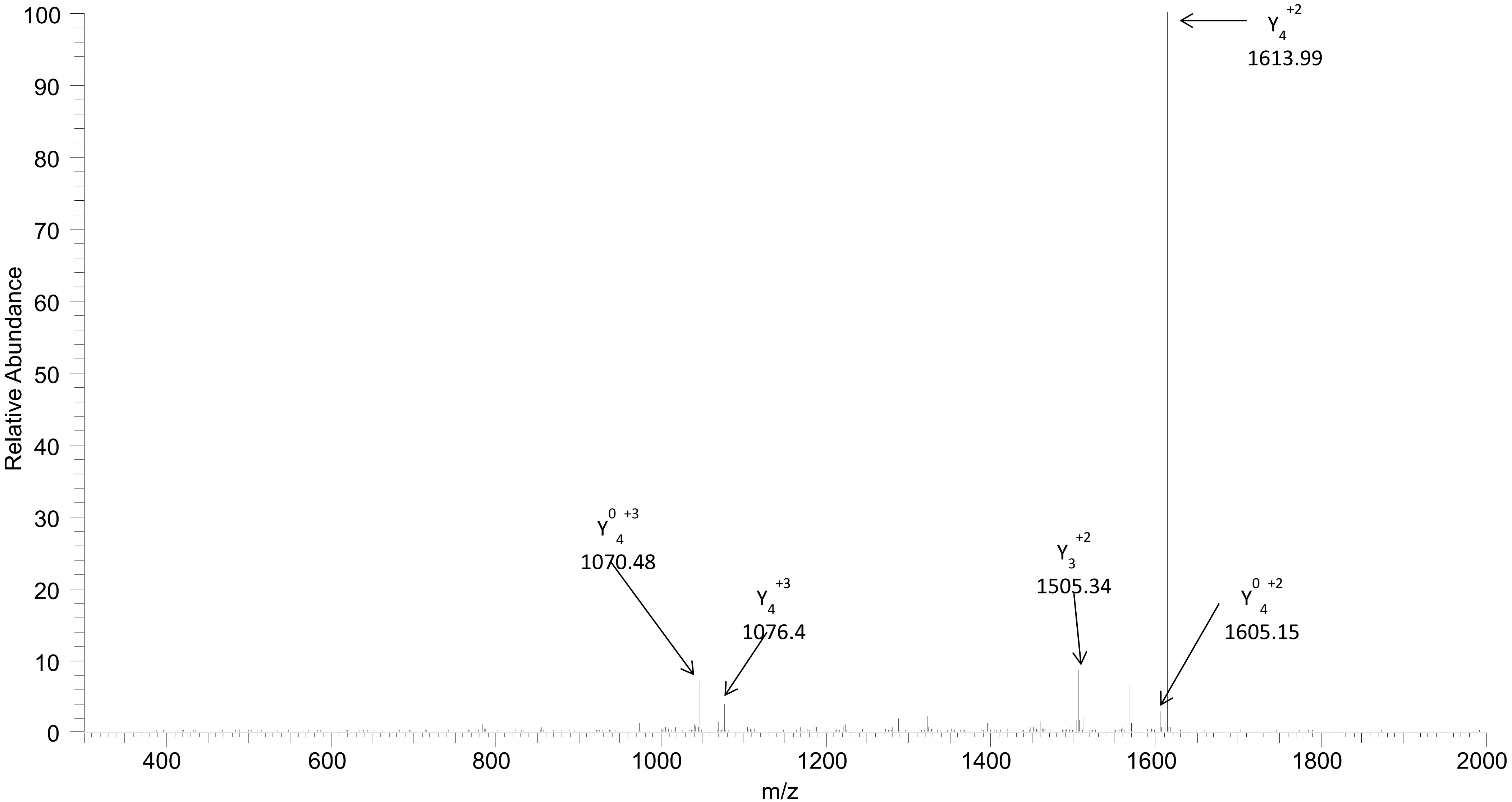

Precursor charge:	6	A H H Y Y Q M L L Q N P K D E A E E K E I Q A L D D T L L L N Y E D D N A T K
Precursor MH+ (Da):	5690.48226	
Peptide mass (MH+):	4634.1758	

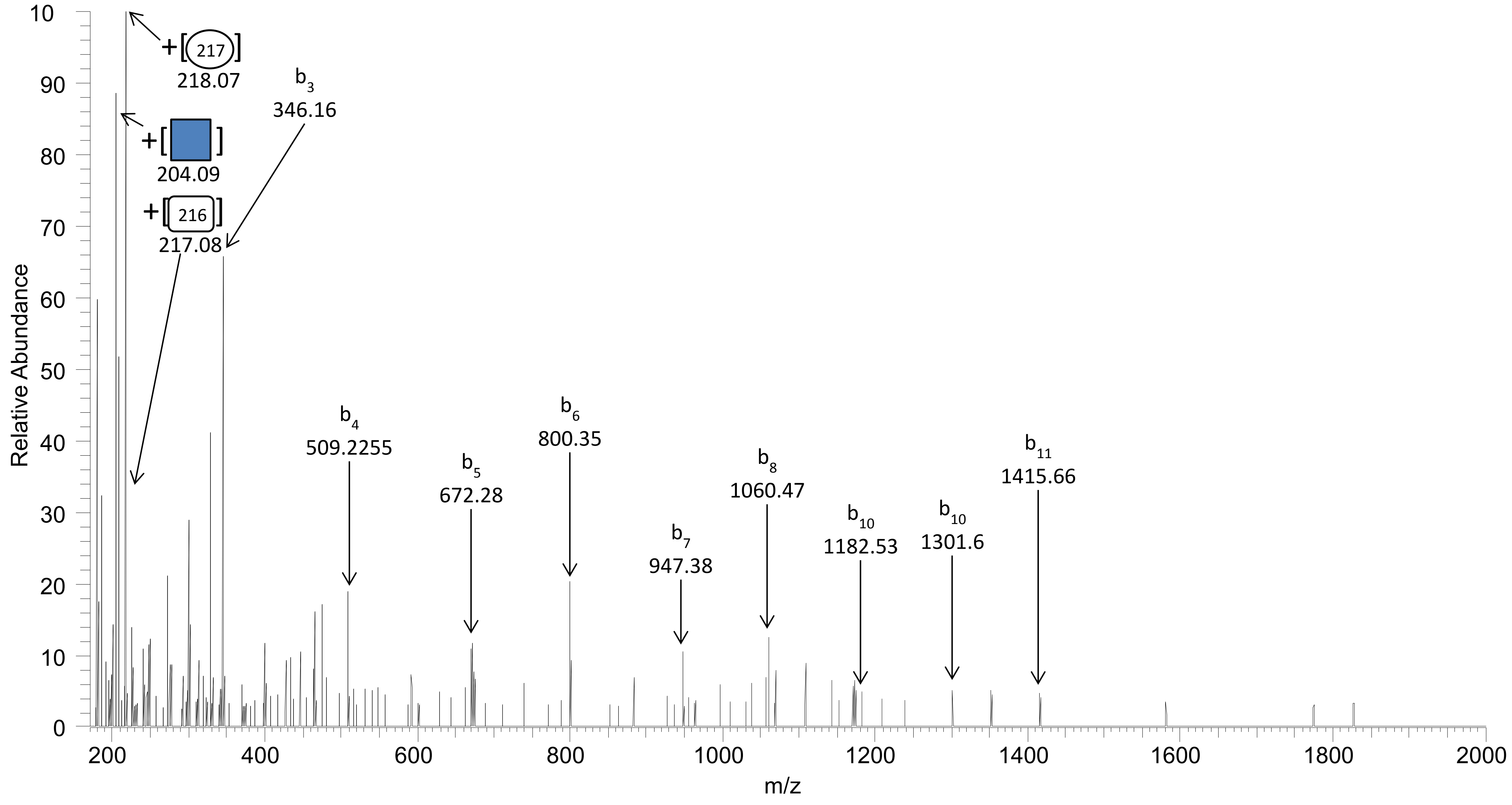

Precursor charge:	6
Precursor MH+ (Da):	5690.48226
Peptide mass (MH+):	4634.1758

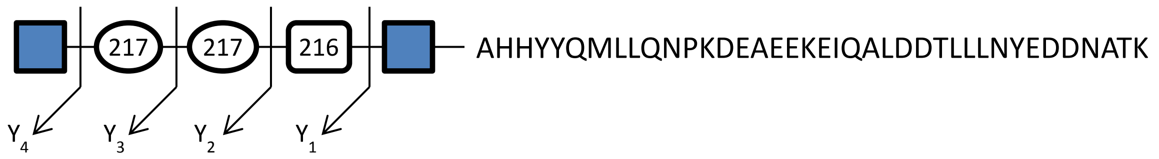

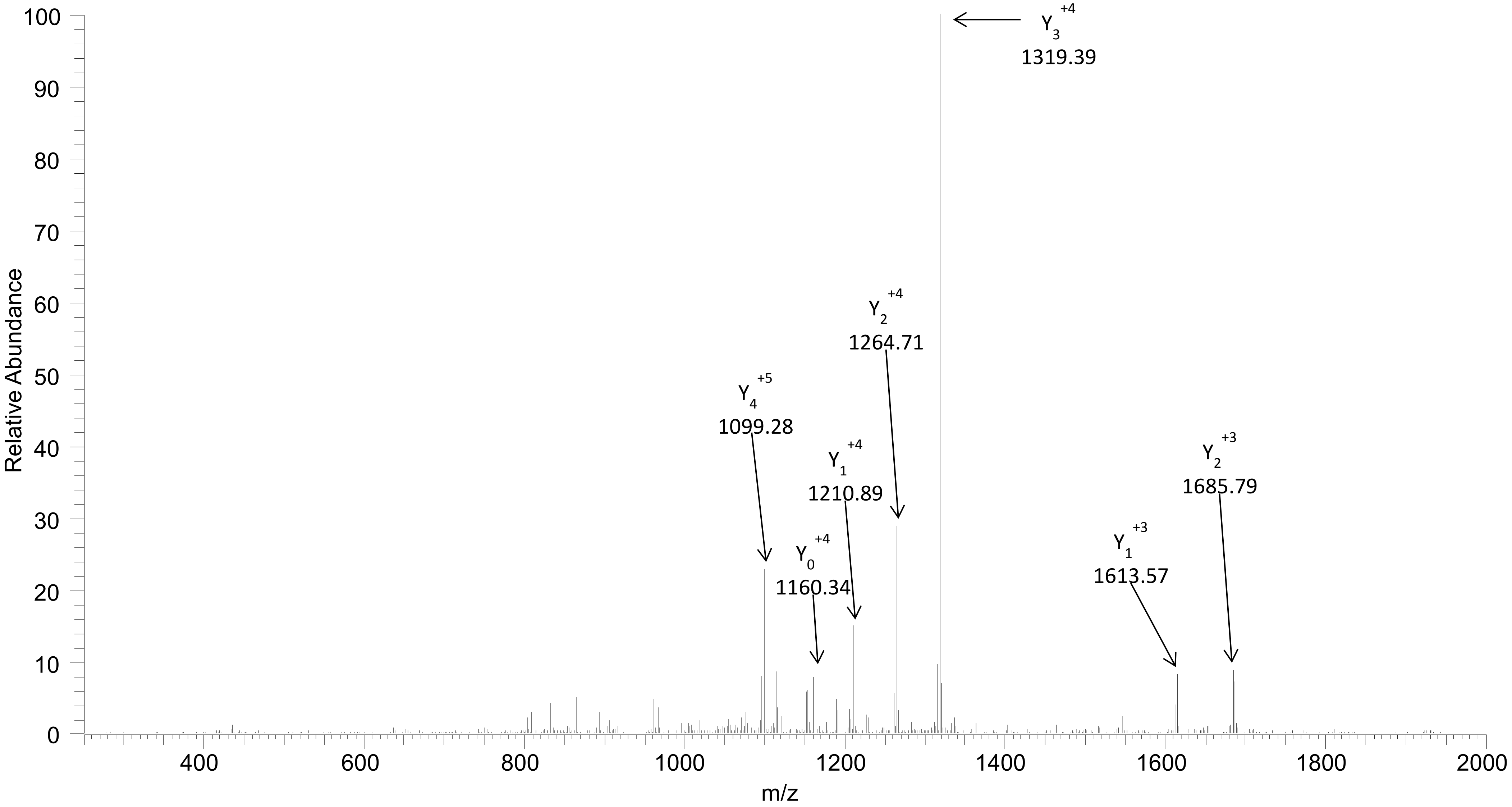

Precursor charge:	3
Precursor MH+ (Da):	2953.26056
Peptide mass (MH+):	1896.9541

K E I P T P N E N N D T K E I R

b2 b3

y13 y12 y11 y10 y7 y6 y5 y4 y3 y2 y1

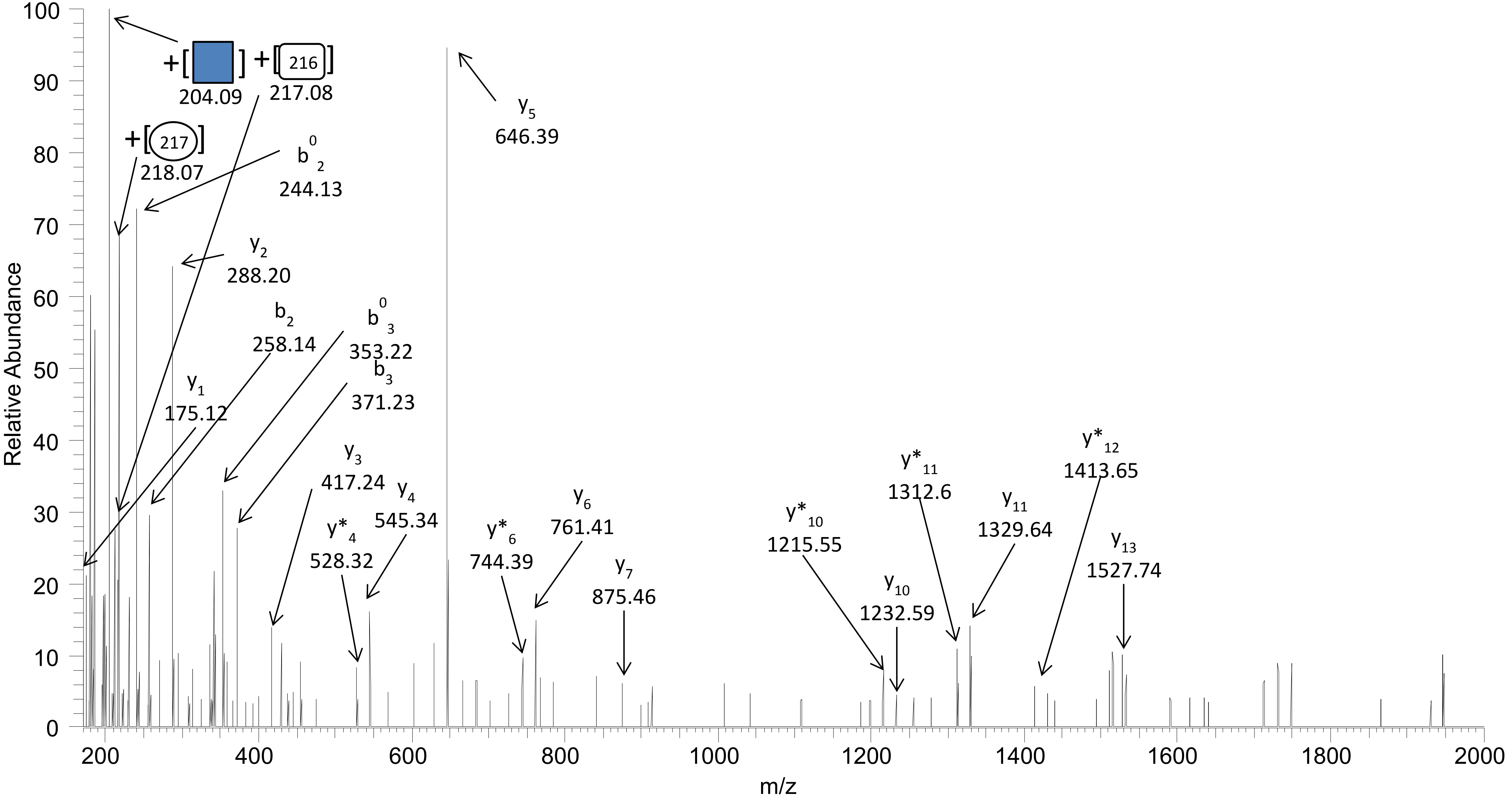

Precursor charge:	3
Precursor MH+ (Da):	2953.26056
Peptide mass (MH+):	1896.9541

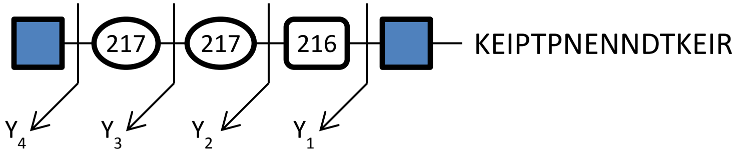

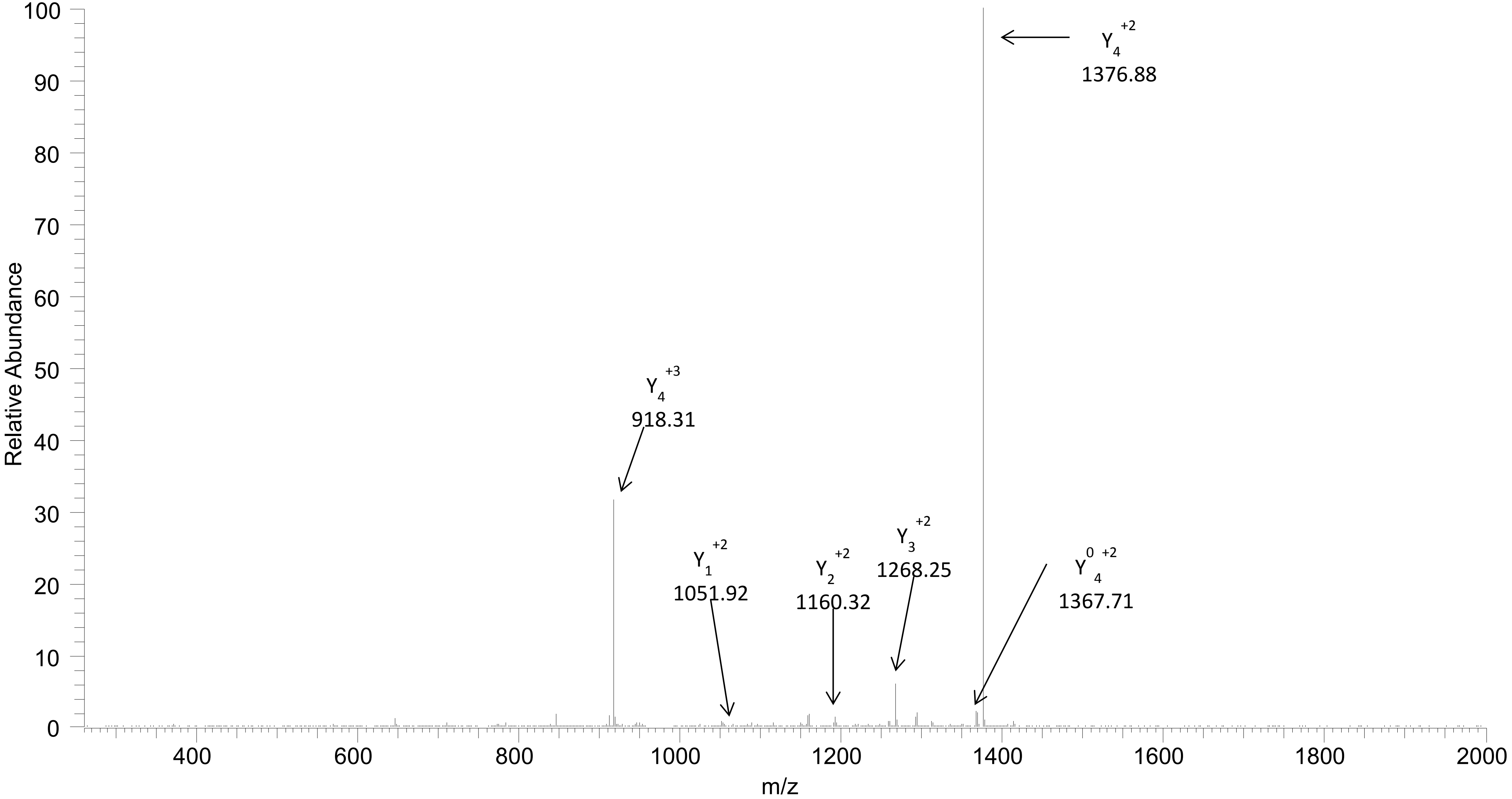

Precursor charge:	6
Precursor MH+ (Da):	5997.85706
Peptide mass (MH+):	4941.5506

T L Q Q E N N Q T S Q S T I P Q I T P P T I S Q E S K P T K P T Q I P S K P K P Q C Q K

(fragment markers: y17, y14, y11, y10, y7, y6, y5, y4, y3)

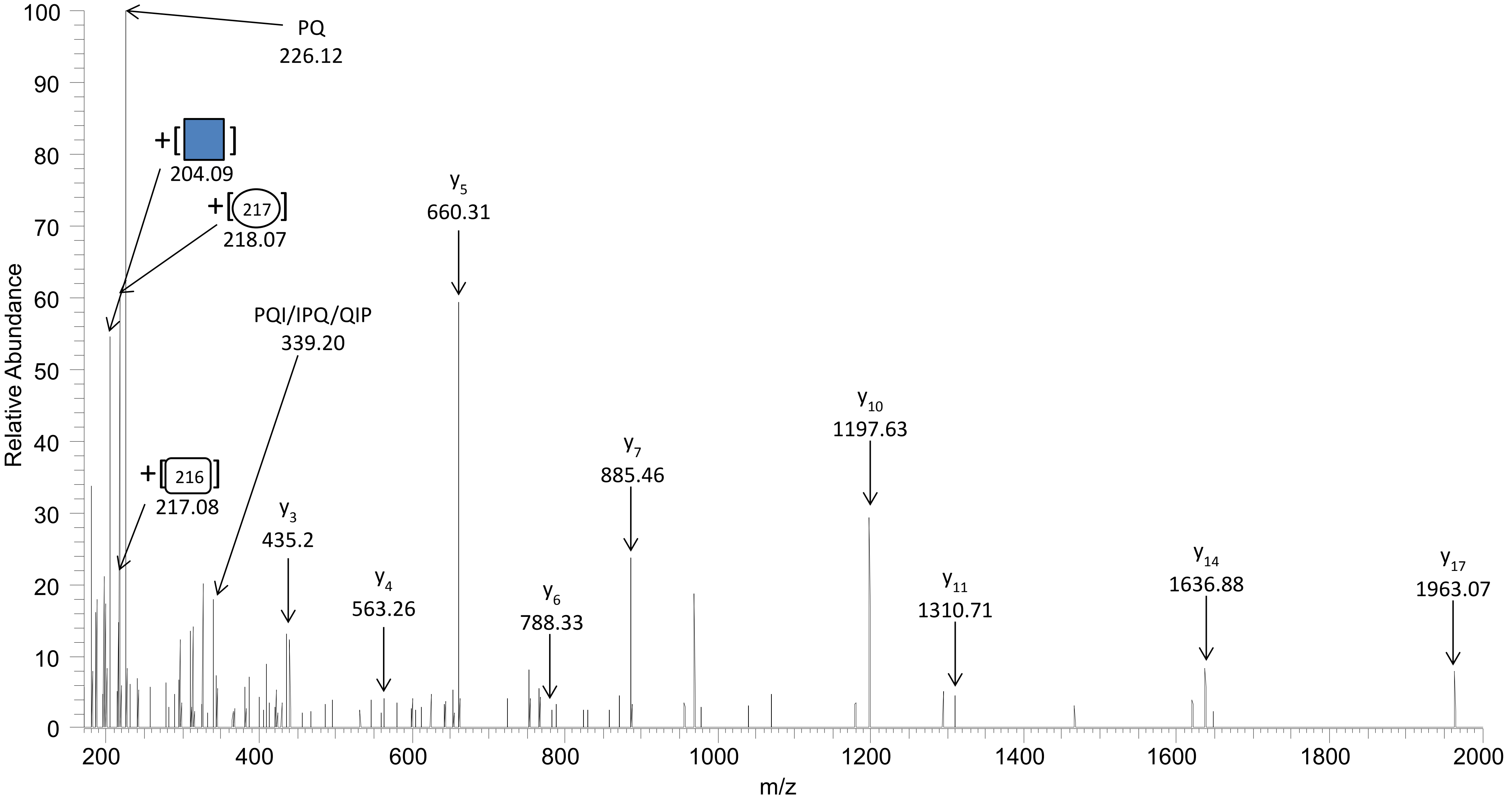

Precursor charge:	6
Precursor MH+ (Da):	5997.85706
Peptide mass (MH+):	4941.5506

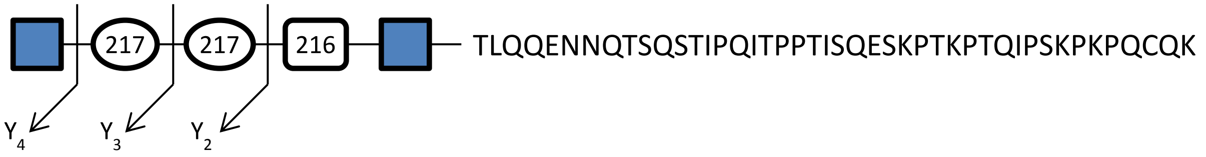

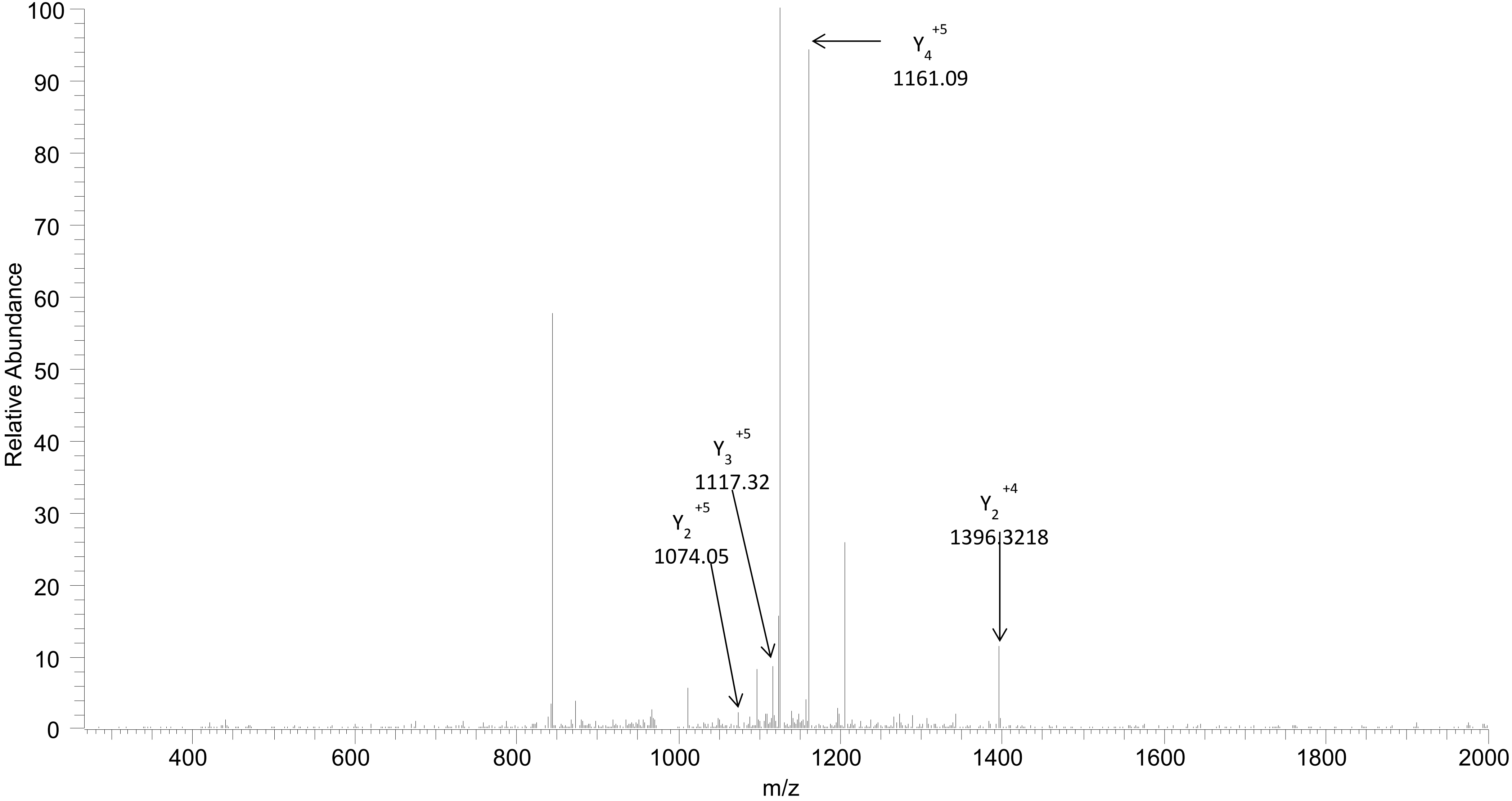

Sup. Fig. S3. CID/HCD MS/MS spectra of identified glycoproteins from *H. pullorum* NCTC 12824 wild-type.

A)

Precursor charge:	4
Precursor MH+ (Da):	4208.96
Peptide mass (MH+):	3152.61

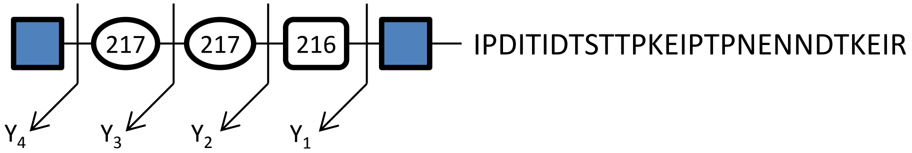

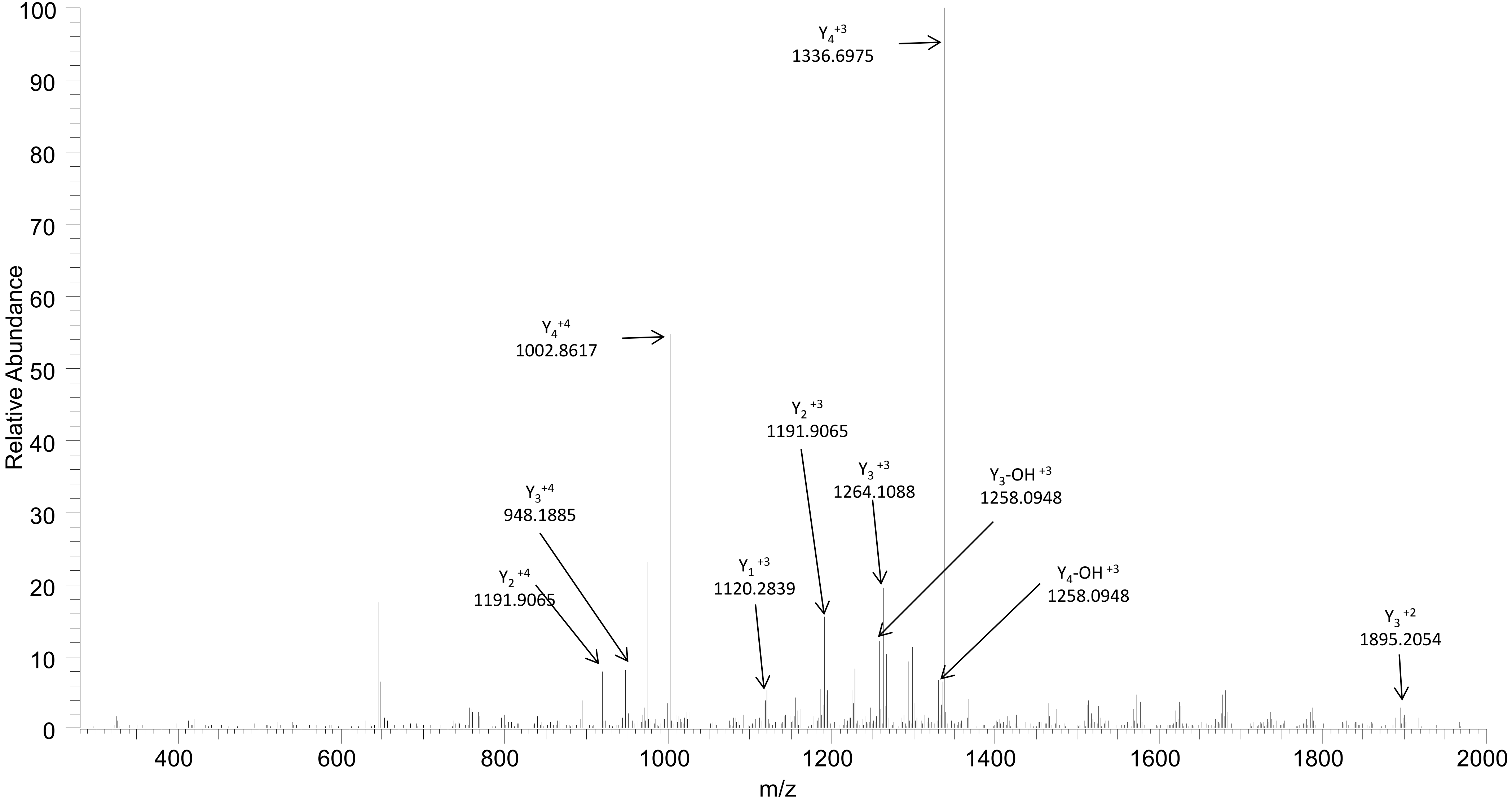

B)

**Precursor charge:**	**4**	I P D I T I D T S T T P K E I P T P N E N N D T K E I R (b2, b3, b4; y6, y5, y4, y3, y2, y1)
**Precursor MH+ (Da):**	4208.96	
**Peptide mass (MH+):**	3152.61	

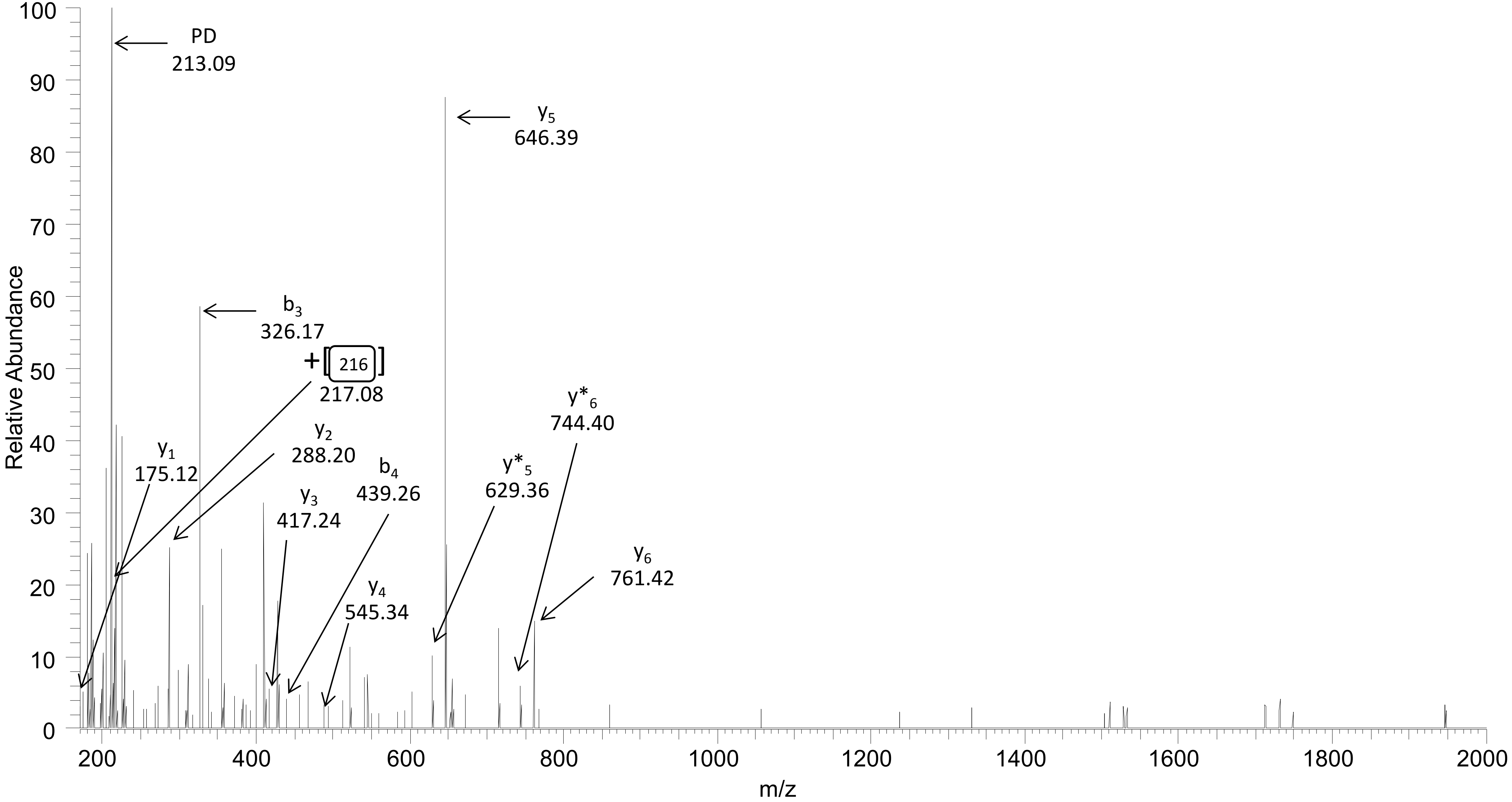

C)

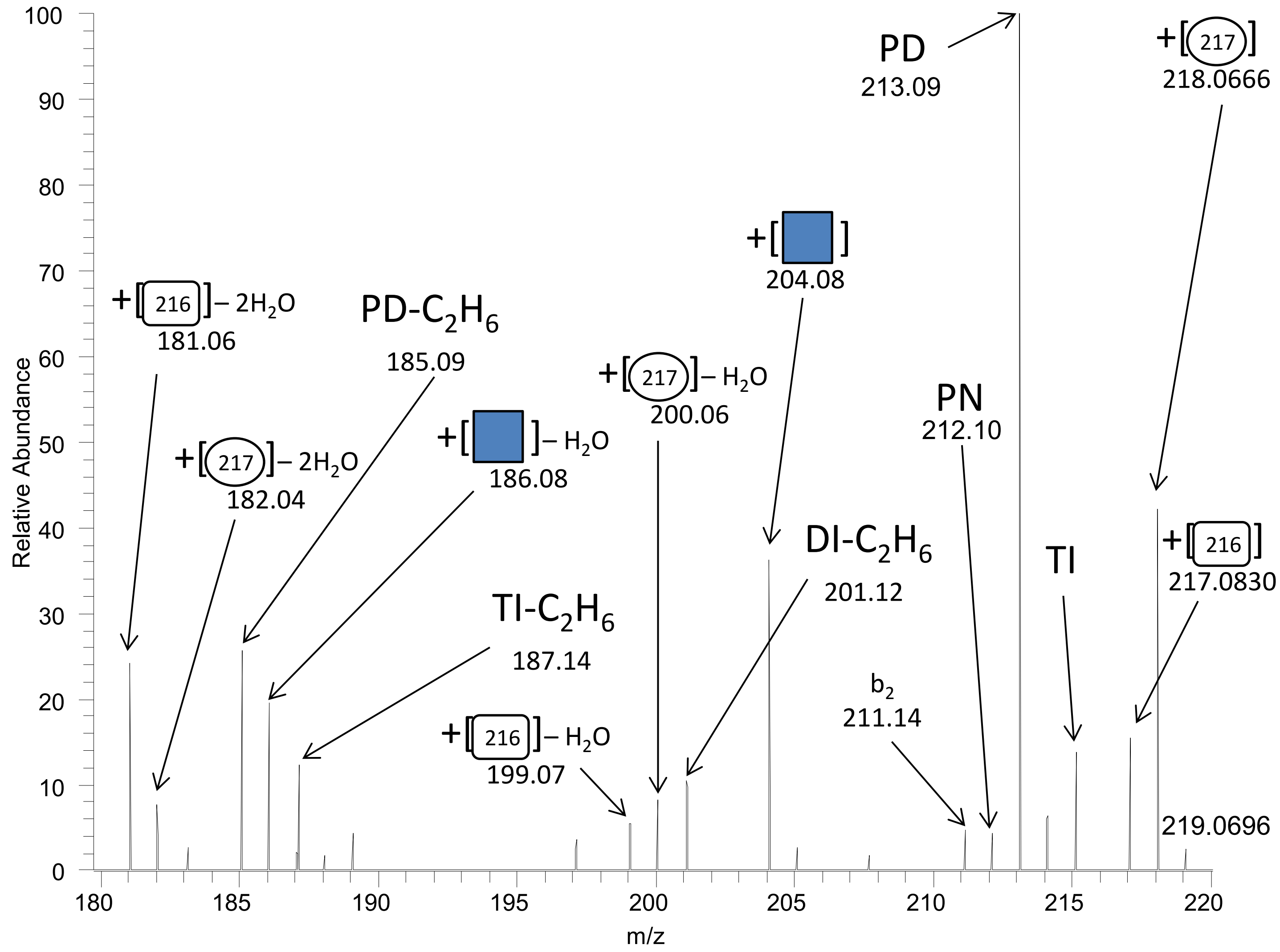

Elemental Composition: **C8 H12 N1 O6**
Monoisotopic M/Z: **218.06591**
Total Abundance: **100.00%**

Isotope Number	m/z	Percent Total	Percent Maximum
0	218.06591	89.67	100.00
1	219.06910	8.72	9.73
2	220.07070	1.48	1.65
3	221.07352	0.12	0.13
4	222.07540	0.01	0.01
5	223.07785	0.00	0.00
6	224.08004	0.00	0.00

Elemental Composition: **C8 H13 N2 O5**
Monoisotopic M/Z: **217.08190**
Total Abundance: **100.00%**

Isotope Number	m/z	Percent Total	Percent Maximum
0	217.08190	89.55	100.00
1	218.08486	9.02	10.07
2	219.08665	1.32	1.48
3	220.08927	0.10	0.11
4	221.09130	0.01	0.01
5	222.09355	0.00	0.00
6	223.09710	0.00	0.00

Sup. Fig. S4. HCD/CID MS/MS spectra for the HgpAhis$_{10}$peptide with mass (MH^+) 4208.96 Da. Peptide sequence: IPDITIDTSTTPKEIPTPNEN**N**DTKEIR A: CID MS/MS spectrumof the identified glycopeptide IPDITIDTSTTPKEIPTPNEN**N**DTKEIR, annotated with respect to fragmentation of the attached glycan B: HCD MS/MS spectrum for the identified glycopeptide IPDITIDTSTTPKEIPTPNEN**N**DTKEIR, annotated with respect to fragmentation of the peptide backbone, C: Close up of peaks contained within the region of 200 and 220 m/z from HCD MS/MS spectrum of IPDITIDTSTTPKEIPTPNEN**N**DTKEIR.

Table SI. Primers used in this study.

Primer	Sequence 5’ to 3’ (restriction sites underlined)
pglA-F	GGT GCT AAA AAC TTC ATC TC
pglA-R	GCC AAA TTG TTC TCT TAA G
pglC-UF	GTT CTG GGA TTG ATA CTG CTC
pglC-UR	GCC AAG CTT AAG TTT GAT GAG
pglC-DF	CAA AAG CTT CCT TGA TGA ACT TCC ACA GC
pglC-DR	GGC TTT GTC TAT TGT TTT GG
pglH-UF	GCA GGC TTG TTT TGC AGG C
pglH-UR	TCA AAG CTT CTG CGC TTA AAA TAT AAG GC
pglH-DF	TTA AAG CTT GGA AGA ATG ATA AAA ACCA
pglH-DR	CTT CTT CGT ATC TAG CAC TC
pglJ-UF	CAA TTC TAA TCA TGC AAT TG
pglJ-UR	TAA AAG CTT TTT TAA ACC GCT TTC
pglJ-DF	TCA AAG CTT AAA GAA TTT CAA AA
pglJ-DR	CTT GGT TTT TGG TGT ATT GG
pglL-UF	GGC TTA GAT TAT GCA AGT GG
pglL-UR	TTT AAG CTT GAG GCA AAT ATG TGG AAT CC
pglL-DF	AAA AAG CTT GGC GTT ATC GTC AAA TCA TC
pglL-DR	CCT CTA AAG TAT CTT TTA CC
wbpO-F	GGC TAG AGA AAT TAA AAA GAT G
wbpO-F	CTT GCT CTT GCA AAA CAA A
wbpS-F	CTC TCC AAA CAC ACA TTA GAG G
wbpS-R	GGG CTT TTG GGA TTC TTG ATA TTT C
wbpSBamHI-F	CTA CCA AAA GGA TCC TAG CAA ATA TTA TC
wbpSBamHI-R	GAT AAT ATT TGC TAG GAT CCT TTT GGT AG
hgpAsphI-F	GAA GCA TGC TGA AAA TTT TAC TAC CAC TGC

hgpABglII-R	ACA AGA TCT GAA GAG TTC AAT CTC TAA GCC
hgpAN46SDM-F	CTT TGG TAT CTT GAT TTT CGT TGG GAG
hgpAN46SDM-F	CTC CCA ACG AAA ATC AAG ATA CCA AAG
Hp23S-F	GTC AGA TGC TGC AGA CCC G
Hp23S-R	GCA GGT AGT CTT CCT GCG ACC
Ery-F	AAG CTT AGG CCG GCC AGT ATA AAA CC
Ery-R	CGA AAA GCT TGG ATC CTT AGG CGC GCC ATC TAC
porAP-F	CAA GAA GGA TCC CTC CTT AAA ATT ACA CGC CT
porAP-R	AAT TCA GGA TCC CAT ATG AAT TCT CCT TGT CAA AAA TTA
hgpABamHI-F	TTT GGA TCC AAC TCT CTA AGT GTA TTA TTT G
hgpABamHI-R	CTC GGA TCC TTA GTG GTG GTG GTG GTG GTG GAA GAG TTC AAT CTC TAA GCC
hgpANdeI-F	AGA ACC CAT ATG AAA ATT TTA CTA CCA CTG C
hgpANdeI-R	AAC TCC ATA TGT TAG TGG TGG TGG TGG TGG TGG TGG TGG TGG TGG AAG AGT TCA TCT CTA AGC C
HppglH-F	GCT CAT ATG CAC ACG CAA ACT AAA AAG ACG
HppglH-R	GGC GGA TCC TTA CTC TTT ATT AAG CAT TAA GC
Cm-F	CAT GAC TAG TAG ATC TCG AGT GCT CGG CGG TGT TCC TTT CCA AG
Cm-R	CAT GAC TAG TGC TAG CCA TGG TTA TTT ATT CAG CAA GTC TTG TAA
*PglBCj*comp-F	GAT CCA TGG GCC TTT GAT AAA GTA AAA ATT GCA C
*PglBCj*comp-R	GCG GCT AGC TTA GTG ATG GTG ATG GTG ATG GTG ATG GTG ATG AAT TTT AAG TTT AAA

	AAC TTT AGC ATC
HppglB1comp-F	GAT CCA TGG AGA TTT TGG ATA CAA TTT GAT AAA
HppglB1comp-R	GCG ACT AGT TTA GTG ATG GTG ATG GTG ATG GTG ATG GTG ATG TTG TTT GAG TTT ATA AAT CG
HppglB2comp-F	GAT CCA TGG GAC TAT TGA CAA GAG ATT CAA GAG TTG
HppglB2comp-R	GCG ACT AGT TTA GTG ATG GTG ATG GTG ATG GTG ATG GTG ATG TTT CCT CAC TTG ATA TAC CAC GAC AAA

Restriction sites are underlined.

Table SII. Plasmids used in this study.

Plasmid name	Description	Source
pCJC1	pUC18 containing Cj0223 fragment interrupted by *cat* cassette	This study
pCJC1pglBCj	pCJC1 with *PglBCj* cloned downstream of *cat* cassette	This study
pCJC1pglBHp1	pCJC1 with *HppglB1* cloned downstream of *cat* cassette	This study
pCJC1pglBHp2	pCJC1 with *HppglB2* cloned downstream of *cat* cassette	This study
pQE*hgpA*	pQE70 containing *H. pullorum hgpA*	This study
pQE*hgpA*N46Q	As pQE*hgpA* with N46Q mutation	This study
pACYCpglB::kan	pACYC184 containing *C. jejuni pgl* locus, *pglB* interrupted with *aphA* cassette	(1)
pMAF10	pMLBAD expressing *PglBCj*	(2)
pMLHpB1	pMLBAD expression *HppglB1*	(3)
pMLHpB2	pMLBAD expression *HppglB2*	(3)
pHPC	pGEM-T Easy containing *H. pullorum* 23rRNA gene (*rrl*) gene fragment	This study
pHPC1	pHPC with ery^{R} cassette interrupting *rrl* fragment	This study
pHPC2	pHPC1 with *C. jejuni porA* promoter region cloned upstream of ery^{R} cassette	This study
pHPC1hgpAhis	pHPC1 with $hgpA\text{-}his_{6}$ cloned downstream of ery^{R} cassette	This study
pHPC2hgpAhis	pHPC2 with $hgpA\text{-}his_{10}$ cloned downstream of *porA* promoter	This study
pHp*pglA*KO	pGEM-T Easy with *H. pullorum pglA* fragment interrupted by *aphA* cassette	This study

pHp*pglC*KO	pGEM-T Easy with *H. pullorum pglC* fragment interrupted by *aphA* cassette	This study
pHp*pglH*KO	pGEM-T Easy with *H. pullorum pglH* fragment interrupted by *aphA* cassette	This study
pHp*pglI*KO	pGEM-T Easy with *H. pullorum pglI* fragment interrupted by *aphA* cassette	This study
pHp*pglJ*KO	pGEM-T Easy with *H. pullorum pglJ* fragment interrupted by *aphA* cassette	This study
pHp*pglL*KO	pGEM-T Easy with *H. pullorum pglL* fragment interrupted by *aphA* cassette	This study
pHp*wbpO*KO	pGEM-T Easy with *H. pullorum wbpO* fragment interrupted by *aphA* cassette	This study
pHp*wbpS*KO	pGEM-T Easy with *H. pullorum wbpS* fragment interrupted by *aphA* cassette	This study
pET15HppglH	pET15b containing *H. pullorum pglH*	This study

Table SIII. Bacterial strains used in this study.

Strain	Parent strain	Relevant genotype	Source
E. coli			
Novablue	*n/a*	*endA1 hsdR17* (r_{K12}^{-} m_{K12}^{+}) *supE44 thi-1 recA1 gyrA96 relA1 lac* F′[*proA*$^{+}$*B*$^{+}$ *lacI*^{q}*Z*Δ*M15*::Tn*10*] (Tet^{R})	Stratagene
H. pullorum			
Hp1	NCTC 12824	Wild-type	
Hp18	Hp1	*pglB1::aphA*	(3)
Hp20	Hp1	*pglA::aphA*	This study
Hp23	Hp1	*pglC::aphA*	This study
Hp21	Hp1	*pglH::aphA*	This study
Hp22	Hp1	*pglJ::aphA*	This study
Hp24	Hp1	*wbpO::aphA*	This study
Hp66	Hp1	*wbpS::aphA*	This study
Hp31	Hp1	23S::*ery*^{R}*hgpA*	This study
Hp67	Hp18	23S::*ery*^{R}*hgpA-his*$_{6}$ *pglB1::aphA*	This study
Hp25	Hp20	23S::*ery*^{R}*hgpA-his*$_{6}$ *pglA::aphA*	This study
Hp26	Hp23	23S::*ery*^{R}*hgpA-his*$_{6}$ *pglC::aphA*	This study
Hp27	Hp21	23S::*ery*^{R}*hgpA-his*$_{6}$ *pglH::aphA*	This study
Hp29	Hp22	23S::*ery*^{R}*hgpA-his*$_{6}$ *pglJ::aphA*	This study
Hp28	Hp24	23S::*ery*^{R}*hgpA-his*$_{6}$ *wbpO::aphA*	This study
Hp68	Hp66	23S::*ery*^{R}*hgpA-his*$_{6}$ *wbpS::aphA*	This study

Hp47	Hp1	23S::*ery*^{R}P_{porA}*hgpA-his*$_{10}$	This study
Hp51	Hp47	23S::*ery*^{R}P_{porA}*hgpA-his*$_{10}$ *pglB1::aphA*	This study
Hp50	Hp47	23S::*ery*^{R}P_{porA}*hgpA-his*$_{10}$ *pglA::aphA*	This study
Hp52	Hp47	23S::*ery*^{R}P_{porA}*hgpA-his*$_{10}$ *pglC::aphA*	This study
Hp53	Hp47	23S::*ery*^{R}P_{porA}*hgpA-his*$_{10}$ *pglH::aphA*	This study
Hp54	Hp47	23S::*ery*^{R}P_{porA}*hgpA-his*$_{10}$ *pglJ::aphA*	This study
Hp55	Hp47	23S::*ery*^{R}P_{porA}*hgpA-his*$_{10}$ *wbpO::aphA*	This study
Hp56	Hp47	23S::*ery*^{R}P_{porA}*hgpA-his*$_{10}$ *wbpS::aphA*	This study
C jejuni			
Cj2	NCTC 11168		
Cj25	Cj2	*pglB*::*aphA*	
Cj124	Cj25	*pglB*::*aphA* *Cj0223*::*catPglBCj*	This study
Cj132	Cj25	*pglB*::*aphA* *Cj0223*::*catHppglB1*	This study
Cj129	Cj25	*pglB*::*aphA* *Cj0223*::*catHppglB2*	This study

Table SIV. Identified H. pullorum NCTC12824 glycoproteins and spectral properties of associated glycopeptides by CID/HCD MS/MS analysis.

Protein Code	Protein ID	Whole Protein Mass (Da)	Observed Mass	Peptide Charge	Precursor MH+ (Da)	Peptide Mass (MH+) (Da)	Mascot Score	Peptide Sequenced	Modifications
Hp01062c	CDS undefined product 1549178:1550728 reverse	56457	1825.7724	3	2882.07796	1825.7715	46.06	^{84}YTDDRVDNGNGDVNGSK^{100}	Deamidated (NQ)
			1826.7555	3	2883.06196	1826.7555	42.36	^{84}YTDDRVDNGNGDVNGSK^{100}	2 Deamidated (NQ)
			1826.7561	3	2883.06196	1826.7555	34.63	^{84}YTDDRVDNGNGDVNGSK^{100}	2 Deamidated (NQ)
			1897.7913	3	2954.09906	1897.7926	27.39	^{84}YTDDRVDNGNGDVNGSKA^{101}	2 Deamidated (NQ)
			1560.6703	3	2616.98296	1560.6765	26.79	^{86}DDRVDNGNGDVNGSK^{100}	
			1561.6602	3	2617.96696	1561.6605	45.85	^{86}DDRVDNGNGDVNGSK^{100}	Deamidated (NQ)
			1562.6446	3	2618.95096	1562.6445	50.58	^{86}DDRVDNGNGDVNGSK^{100}	2 Deamidated (NQ)
			1561.6611	3	2617.96696	1561.6605	28.37	^{86}DDRVDNGNGDVNGSK^{100}	Deamidated (NQ)
			1446.6324	3	2502.93996	1446.6335	51.7	^{87}DRVDNGNGDVNGSK^{100}	Deamidated (NQ)
			1447.6166	3	2503.92396	1447.6175	51.73	^{87}DRVDNGNGDVNGSK^{100}	2 Deamidated (NQ)
			1446.6332	3	2502.93996	1446.6335	30.8	^{87}DRVDNGNGDVNGSK^{100}	Deamidated (NQ)
			1447.6162	3	2503.92396	1447.6175	63.45	^{87}DRVDNGNGDVNGSK^{100}	2 Deamidated (NQ)
			1517.6711	3	2573.97706	1517.6706	35.63	^{87}DRVDNGNGDVNGSKA^{101}	Deamidated (NQ)
			1330.6226	3	2386.92906	1330.6226	50.32	^{88}RVDNGNGDVNGSK^{100}	
			1331.6063	3	2387.91306	1331.6066	51.23	^{88}RVDNGNGDVNGSK^{100}	Deamidated (NQ)
			1331.6072	2	2387.91306	1331.6066	36.79	^{88}RVDNGNGDVNGSK^{100}	Deamidated (NQ)
			1332.5881	3	2388.89706	1332.5906	45.23	^{88}RVDNGNGDVNGSK^{100}	2 Deamidated (NQ)
			1332.5896	2	2388.89706	1332.5906	27.06	^{88}RVDNGNGDVNGSK^{100}	2 Deamidated (NQ)
			1402.643	3	2458.95016	1402.6437	38.2	^{88}RVDNGNGDVNGSKA^{101}	Deamidated (NQ)
			1403.6273	3	2459.93416	1403.6277	37.29	^{88}RVDNGNGDVNGSKA^{101}	2 Deamidated (NQ)
			1402.645	3	2458.95016	1402.6437	54.1	^{88}RVDNGNGDVNGSKA^{101}	Deamidated (NQ)
			1403.6259	3	2459.93416	1403.6277	38.11	^{88}RVDNGNGDVNGSKA^{101}	2 Deamidated (NQ)
Hp00296c	CDS undefined product 408023:408979 reverse	35661	1944.9364	3	3001.24526	1944.9388	24.96	^{159}KKDTIKEDSVENNGSAPN^{176}	
			2015.9767	4	3072.28236	2015.9759	58.8	^{159}KKDTIKEDSVENNGSAPN^{176}	
			2015.9754	3	3072.28236	2015.9759	23.45	^{159}KKDTIKEDSVENNGSAPN^{176}	

			2201.0555	3	3257.36246	2201.056	25.62	159KKDTIKEDSVENNGSAPNANA179	
			2201.0548	4	3257.36246	2201.056	41.85	159KKDTIKEDSVENNGSAPNANA179	
			2499.225	4	3555.52656	2499.2201	54.98	159KKDTIKEDSVENNGSAPNANANIA182	
			1390.658	3	2446.96406	1390.6576	33.01	160KDTIKEDSVENN171	
			1887.8814	3	2944.18746	1887.881	56.75	160KDTIKEDSVENNGSAPNA177	
			1887.8831	2	2944.18746	1887.881	26.47	160KDTIKEDSVENNGSAPNA177	
			1888.8647	3	2945.17146	1888.865	69.87	160KDTIKEDSVENNGSAPNA177	Deamidated (NQ)
			1887.8809	3	2944.18746	1887.881	25.18	160KDTIKEDSVENNGSAPNA177	
			1888.8667	3	2945.17146	1888.865	24.38	160KDTIKEDSVENNGSAPNA177	Deamidated (NQ)
			2001.9259	3	3058.23036	2001.9239	43.75	160KDTIKEDSVENNGSAPNAN178	
			2073.9448	3	3130.25146	2073.945	55.34	160KDTIKEDSVENNGSAPNANA179	Deamidated (NQ)
			2187.0051	3	3243.31036	2187.0039	47.01	160KDTIKEDSVENNGSAPNANAN180	
			2187.9884	3	3244.29436	2187.9879	54.64	160KDTIKEDSVENNGSAPNANAN180	Deamidated (NQ)
			2371.1308	3	3427.43156	2371.1251	36.36	160KDTIKEDSVENNGSAPNANANIA182	
			2372.1082	3	3428.41556	2372.1091	54.18	160KDTIKEDSVENNGSAPNANANIA182	Deamidated (NQ)
			3486.6388	4	4542.94106	3486.6346	55.57	160KDTIKEDSVENNGSAPNANANIATIESAENNQK192	Deamidated (NQ)
			3486.6491	4	4542.94106	3486.6346	21.97	160KDTIKEDSVENNGSAPNANANIATIESAENNQK192	Deamidated (NQ)
			3485.6603	4	4541.95696	3485.6505	48.67	160KDTIKEDSVENNGSAPNANANIATIESAENNQK192	
			3486.6505	4	4542.94106	3486.6346	58.6	160KDTIKEDSVENNGSAPNANANIATIESAENNQK192	Deamidated (NQ)
			3830.8267	5	4887.11056	3830.8041	25.5	160KDTIKEDSVENNGSAPNANANIATIESAENNQKESK195	Deamidated (NQ)
			3830.8157	5	4887.11056	3830.8041	54.53	160KDTIKEDSVENNGSAPNANANIATIESAENNQKESK195	Deamidated (NQ)
			3357.567	4	4413.86206	3357.5556	21.9	161DTIKEDSVENNGSAPNANANIATIESAENNQK192	
			3357.5507	3	4413.86206	3357.5556	21.45	161DTIKEDSVENNGSAPNANANIATIESAENNQK192	
			3701.7179	4	4758.03166	3701.7252	28.01	161DTIKEDSVENNGSAPNANANIATIESAENNQKESK195	
			2371.1319	3	3427.43156	2371.1251	27.25	175PNANANIATIESAENNQKESKQ196	Deamidated (NQ)
Hp00314c	CDS undefined product 438888:441281 reverse	93678	4635.2057	4	5691.46626	4635.1598	21.4	427AHHYYQMLLQNPKDEAEEKEIQALDDTLLLNYEDDNATK465	Deamidated (NQ); Oxidation (M)
			4634.1816	6	5690.48226	4634.1758	46.17	427AHHYYQMLLQNPKDEAEEKEIQALDDTLLLNYEDDNATK465	Oxidation (M)
			4634.1818	4	5690.48226	4634.1758	23.28	427AHHYYQMLLQNPKDEAEEKEIQALDDTLLLNYEDDNATK465	Oxidation (M)

			4634.1769	5	5690.48226	4634.1758	42.41	^{427}AHHYYQMLLQNPKDEAEEKEIQALDDTLLLNYEDDNATK^{465}	Oxidation (M)
			4790.2878	6	5846.58336	4790.2769	21.72	^{427}AHHYYQMLLQNPKDEAEEKEIQALDDTLLLNYEDDNATKR^{466}	Oxidation (M)
			4790.2847	5	5846.58336	4790.2769	29.08	^{427}AHHYYQMLLQNPKDEAEEKEIQALDDTLLLNYEDDNATKR^{466}	Oxidation (M)
Hp00510	CDS undefined product 737534:738265 forward	28021	1896.9537	4	2953.26056	1896.9541	49.66	^{44}KEIPTPNENNDTKEIR^{59}	
			1896.9534	3	2953.26056	1896.9541	56.58	^{44}KEIPTPNENNDTKEIR^{59}	
			1897.9391	4	2954.24456	1897.9381	43.65	^{44}KEIPTPNENNDTKEIR^{59}	Deamidated (NQ)
			1370.6324	2	2426.93786	1370.6314	27.51	^{45}EIPTPNENNDTK^{56}	
Hp00561c	CDS undefined product 817606:818622 reverse	38588	4941.5575	6	5997.85706	4941.5506	37.78	^{68}TLQQENNQTSQSTIPQITPPTISQESKPTKPTQIPSKPKPQCQK^{111}	
			4943.5028	6	5999.82506	4943.5186	24.5	^{68}TLQQENNQTSQSTIPQITPPTISQESKPTKPTQIPSKPKPQCQK^{111}	2 Deamidated (NQ)

References

(1) **Linton D, Dorrell N, Hitchen PG, Amber S, Karlyshev AV, Morris HR, Dell A, Valvano MA, Aebi M, Wren BW.** 2005. Functional analysis of the *Campylobacter jejuni* N-linked protein glycosylation pathway. Mol Microbiol **55:**1695-703.

(2) **Feldman MF, Wacker M, Hernandez M, Hitchen PG, Marolda CL, Kowarik M, Morris HR, Dell A, Valvano MA, Aebi M.** 2005. Engineering N-linked protein glycosylation with diverse O antigen lipopolysaccharide structures in *Escherichia coli*. Proc Natl Acad Sci U S A **102:**3016-21.

(3) **Jervis, AJ, Langdon R, Hitchen P, Lawson AJ, Wood AG, Fothergill JL, Morris JL, Dell A, Wren BW, Linton D.** 2010. Characterization of N-linked protein glycosylation in *Helicobacter pullorum*. J Bacteriol **192:**5228-36.
